# Design Strategies for Carbon Nanotube-Based Biosensors in Complex Biological Media: From Interfacial Physics to System-Level Translation

**DOI:** 10.3390/s26217060

**Published:** 2026-11-08

**Authors:** Jongwan Lee, Chang-Ho Han, Bon Joon Koo, Minseok Kim

**Affiliations:** 1 School of Mechanical Engineering, Kunsan National University, Gunsan 54150, Republic of Korea; jongwanlee@kunsan.ac.kr; 2 Advanced Machinery Technology Research Institute, Kunsan National University, Gunsan 54150, Republic of Korea; 3 Korea Institute of Science and Technology Europe Forschungsgesellschaft mbH, 66123 Saarbrücken, Germany; chang-ho.han@kist-europe.de; 4 Zero9 Material Co., Ltd., Jeonju 54852, Republic of Korea; koo@zero9.co.kr; 5 School of Mechanical Engineering, Kumoh National Institute of Technology, Gumi 39177, Republic of Korea; 6 Department of Aeronautics, Mechanical and Electronic Convergence Engineering, Kumoh National Institute of Technology, Gumi 39177, Republic of Korea

**Keywords:** carbon nanotube, biosensor, CNT-FET, SWCNT, complex biological media, biofouling, microfluidics, near-infrared fluorescence, molecular recognition, reproducibility

## Abstract

**Highlights:**

**What are the main findings?**

- Design principles for carbon nanotube biosensors operating in complex biological media are reviewed, with emphasis on receptor organization, corona-phase control, and matrix-dependent signal modulation.

**What are the implications of the main findings?**

- Recent advances in electrical, optical, microfluidic, and data-driven CNT sensing strategies are compared to identify approaches for improving selectivity, stability, and in vivo applicability.

**Abstract:**

Carbon nanotube (CNT) biosensors offer sensitive electrical and near-infrared optical readouts, yet performance in defined buffers does not necessarily translate to reliable measurements in complex biological media. Ionic screening, nonspecific adsorption, variable sample composition, and transport limitations can disrupt the relationship between molecular recognition and sensor output. This review examines how interfacial engineering and system-level design address these constraints across serum, plasma, whole blood, saliva, urine, and intracellular or tissue environments. We connect receptor orientation and dimensions, antifouling coatings, and designed corona phases to their effects on analyte accessibility, transduction, and signal stability. We then discuss sample separation, enrichment, and microfluidic delivery as integral parts of the measurement workflow. Electrical CNT architectures and optical single-walled CNT sensors are compared in terms of their operating mechanisms, matrix-dependent limitations, and validation requirements. Particular attention is given to distinguishing direct sensing from CNT-assisted sample preparation, optical visibility from selective analyte measurement, and fortified-sample testing from clinical validation. We also examine data-driven recognition discovery, manufacturing reproducibility, and reporting practices needed for meaningful comparison across platforms. The central design principle is to optimize the complete sample-to-answer process around the dominant limitation in the intended biological environment. Progress toward practical CNT biosensing therefore requires matrix-matched calibration, explicit characterization of sample conditioning, and independent validation across devices, batches, and biological specimens.

## 1. Introduction

### 1.1. Why CNT Biosensors Remain Attractive

Carbon nanotubes (CNTs) remain attractive biosensing materials because they bring molecular recognition into close contact with an electrically or optically responsive nanoscale interface. Their high surface-to-volume ratio provides an extensive interface for functionalization, while their electrical properties support several routes for converting interactions at that interface into measurable signals. The signal-conversion mechanism depends on the CNT platform. In CNT-FETs and chemiresistive devices, molecular binding can change local electrostatic potential, carrier scattering, contact injection, or the resistance of CNT-FET junctions. In CNT-containing electrochemical electrodes, the measured response can instead arise from interfacial electron transfer, capacitive charging, diffusion through a porous CNT layer, or changes in the electrode–electrolyte interface. In optical SWCNT sensors, molecular interactions can modify fluorescence intensity or wavelength through corona-phase rearrangement, quenching, or changes in the local dielectric environment. These mechanisms should therefore not be treated as interchangeable consequences of the high surface-to-volume ratio [1]. CNTs can therefore serve as transducing channels, components of electrochemical electrodes, or scaffolds for recognition elements. These roles are related, but they impose different requirements on material selection, surface chemistry, and measurement configuration [2].

In electrical devices, the low-dimensional conducting channel makes surface-associated perturbations relevant to charge transport. This feature motivates label-free detection, but the measured response depends on how recognition is coupled to the device. Compact junction and network-transistor architectures further demonstrate the scope for miniaturized CNT devices [3,4]. Compatibility with flexible formats is also attractive, although mechanical adaptability and small device dimensions do not by themselves establish reliable assay performance [2,5].

Semiconducting single-walled CNTs (SWCNTs) provide a complementary optical route through near-infrared fluorescence. Their emission can be coupled to molecular recognition by engineering the surrounding corona phase, as demonstrated by protein-targeted corona phase molecular recognition (CoPhMoRe) and subsequent optical uric acid detection [6,7]. Optical interrogation is particularly relevant where electrical contacts or electrolyte-gating arrangements are difficult to implement. Fluorescent CNTs have also enabled imaging in biological organisms, although the ability to visualize nanotube localization must be distinguished from selective measurement of an analyte [8,9]. The appeal of CNTs thus lies in a versatile set of material and transduction options. Realizing that versatility requires matching each option to the biological environment in which the measurement is intended to operate.

CNT-based biosensors are not limited to antibody-based immunosensors. Different recognition formats have been implemented according to the target and transduction mechanism, including aptamers for protein and small-molecule detection [10,11,12], nucleic-acid probes for DNA hybridization [13,14,15,16], affinity- or enzyme-related recognition systems [17,18], and designed corona phases for optical molecular recognition [6,7,19,20,21,22]. Phage-based recognition has also been incorporated into CNT-containing impedimetric platforms for pathogen detection [23]. These recognition elements are not interchangeable: their size, orientation, binding mechanism, and distance from the CNT interface determine target accessibility, matrix susceptibility, and coupling to the electrical or optical signal. Accordingly, this review uses “CNT-based biosensors” as an inclusive term covering multiple recognition chemistries and transduction formats rather than immunosensors alone.

**Figure 1:**
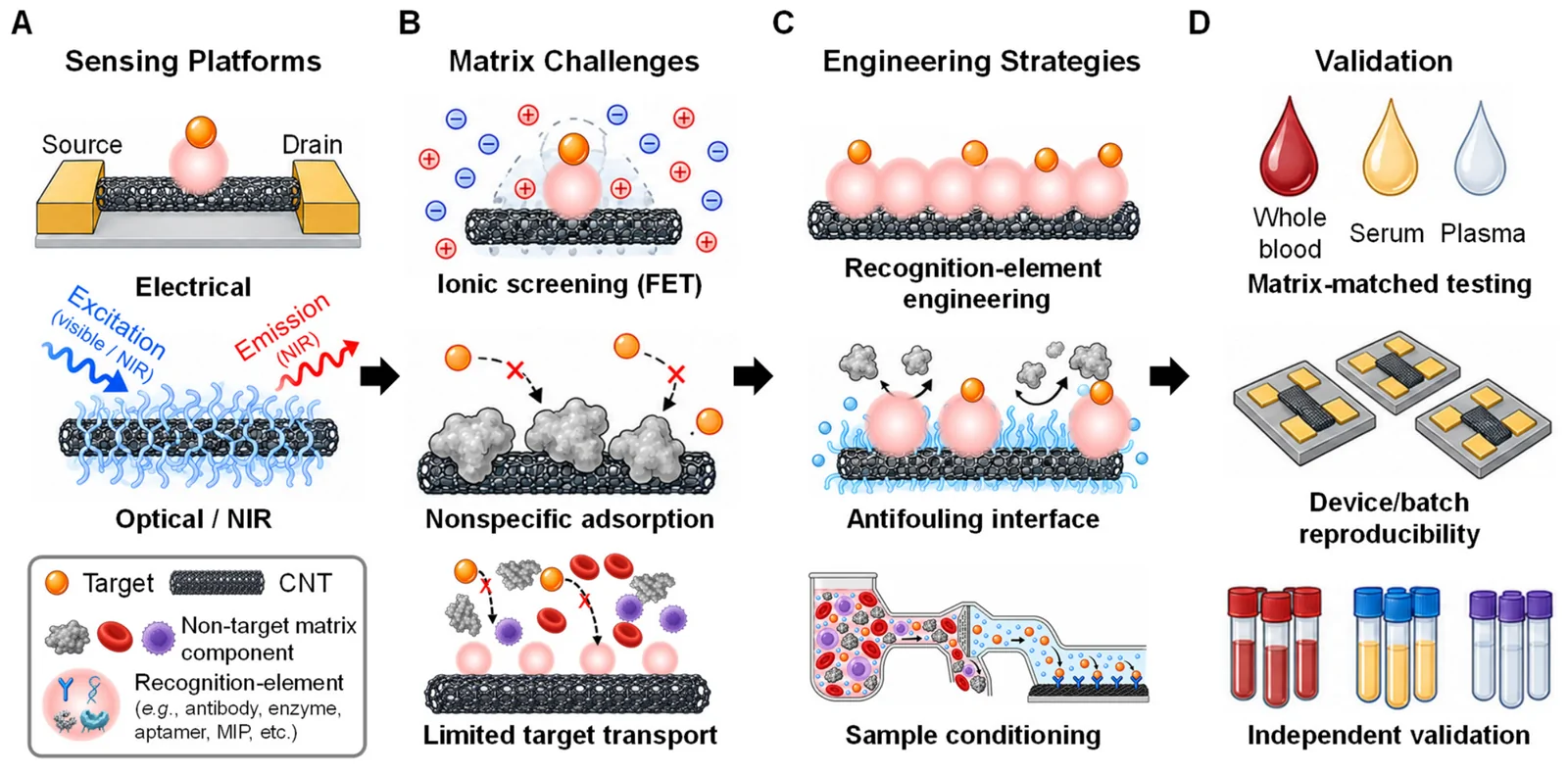
Design framework for CNT biosensors in complex biological media. (**A**) Electrical and near-infrared (NIR) optical sensing platforms. (**B**) Matrix-dependent challenges, including ionic screening in field-effect transistors, nonspecific adsorption, restricted target transport, and corona stability and optical-background effects. (**C**) Recognition-element engineering, antifouling interfaces, and sample conditioning to improve sensor access and performance. The recognition elements shown schematically may include antibodies, enzymes, aptamers, nucleic-acid probes, molecularly imprinted polymers, and other target-specific recognition chemistries. (**D**) Validation through matrix-matched testing, device and batch reproducibility, and independent evaluation. The schematic is conceptual and is not drawn to scale.

Figure 1 summarizes the electrical and optical transduction routes (A), matrix-dependent constraints (B), interface and sample-conditioning strategies (C), and validation requirements (D).

### 1.2. Why Excellent Analytical Sensitivity Has Not Guaranteed Translation

Successful molecular recognition does not necessarily produce a measurable electrical response. Receptor geometry and the measurement environment determine how strongly binding couples to the CNT channel [24]. Buffer exchange or dilution may improve this coupling, but the resulting performance describes the complete assay workflow rather than direct measurement in the original sample.

García-Hevia et al. examined how exposure to serum altered CNT-associated protein layers and found different protein profiles for samples from different human donors [25]. Their findings provide a biological counterpart to the electrical constraint in Figure 1B, showing that nominally identical CNTs can encounter different adsorbed environments. The engineering response in Figure 1C is therefore to coordinate receptor presentation, protection against nonspecific adsorption, and sample conditioning. A deliberately designed recognition corona, such as that investigated by Bisker et al., serves a different function from an uncontrolled protein layer and must be assessed accordingly [6].

Serum, plasma, saliva, urine, sweat, whole blood, and interstitial fluid constitute distinct measurement environments; intracellular and in vivo operation add requirements for access, localization, and calibration. Two studies illustrate the value and limits of matrix-specific testing. Yoon et al. compared optical uric acid responses in water and pooled human urine and examined spiked urine [7], whereas Zamzami et al. measured recombinant SARS-CoV-2 S1 in fortified saliva using a carbon nanotube field-effect transistor (CNT-FET) [26]. These experiments support the matrix-matched evaluation represented in Figure 1D. They do not establish patient-level diagnostic accuracy. The two studies also differ in both transduction mode and validation level: Yoon et al. used an optical SWCNT corona-phase sensor and evaluated pooled and spiked urine, whereas Zamzami et al. used an antibody-functionalized CNT-FET to measure recombinant S1 in fortified saliva. Thus, these studies demonstrate matrix-relevant analytical testing but not diagnostic performance in untreated individual specimens. Likewise, detecting human serum albumin in a prepared solution is different from measuring it in serum [4], and observing fluorescent CNTs in an organism is different from quantifying a selectively recognized analyte [8,9].

The analytical challenges differ substantially among biological matrices. Serum contains abundant proteins that can form a protein corona on CNT surfaces, potentially altering receptor accessibility and nonspecific adsorption [25]. Plasma presents similar protein-related challenges, but the composition of the processed sample can also depend on the separation protocol. These factors should be reported because they may influence the matrix presented to the CNT sensor.

Whole blood is more demanding because cells, hemoglobin, platelets, clotting factors, and high viscosity can obstruct microfluidic transport, increase optical background, foul exposed surfaces, and produce heterogeneous sample delivery. For whole-blood operation, plasma extraction, filtration, cell separation, or controlled dilution is often more appropriate than direct exposure of an unprotected CNT interface [27,28,29,30]. Saliva generally contains less protein than serum or plasma but includes mucins, enzymes, cells, microorganisms, and variable salt concentrations. These components can restrict target transport and produce nonspecific adsorption and sample-to-sample variation. Dilution, filtration, mucin-tolerant delivery, and antifouling interfaces with sufficient analyte accessibility are therefore relevant to salivary sensing [26,28,31]. In this context, the study by Zamzami et al. demonstrated CNT-FET detection of recombinant SARS-CoV-2 S1 in fortified saliva, but it did not establish performance in untreated clinical saliva [26].

Urine has a generally lower protein background than serum or plasma but exhibits substantial variation in pH, ionic strength, osmolality, and metabolite composition. These factors can alter Debye screening, receptor charge state, electrical baseline, CNT fluorescence, and apparent selectivity [7,32,33]. Urine assays therefore require pH- and ionic-strength-aware calibration, controlled dilution or targeted pretreatment, and validation across the relevant matrix and concentration ranges. The optical uric-acid study by Yoon et al. compared water, pooled urine, and spiked urine, with ascorbate-oxidase pretreatment, and should therefore be interpreted as matrix-relevant analytical testing rather than clinical diagnostic validation.

These differences imply that no single CNT surface treatment is optimal for all biological matrices. Serum and plasma generally favor hydrated antifouling layers, controlled receptor orientation, and matrix-matched calibration. Whole blood additionally requires upstream separation or enrichment and microfluidic control of cell transport. Saliva requires delivery and surface strategies that tolerate mucins and variable viscosity, whereas urine requires explicit control or measurement of pH and ionic strength. In all cases, the dominant limitation—biofouling, ionic screening, transport, optical background, or target recovery—should be identified and evaluated as part of the complete sample-to-answer workflow.

### 1.3. Scope and Organizing Principle

Previous reviews have broadly surveyed CNT-based biosensors according to nanotube type, device architecture, recognition element, or transduction mode [34,35,36,37]. The present review instead focuses on how interfacial design and system-level assay configuration jointly determine sensor performance in complex biological media. The discussion first addresses electrostatic coupling, recognition-layer geometry, and biointerface formation, and then considers transport, separation, enrichment, dilution, and microfluidic delivery before comparing electrical, electrochemical, and optical CNT-containing architectures. The role of the nanotube is kept explicit throughout. CNT channel walls used for plasma separation constitute a sample-preparation module, with albumin measured by a separate downstream assay [27]. CNT-assisted virus capture followed by gold-assisted Raman identification represents another architecture and should not be conflated with CNT field-effect sensing or SWCNT near-infrared fluorescence [38]. These examples illustrate why CNT biosensors should be compared through the complete sample-to-answer process rather than by sensitivity alone. The key perspective of this review is that CNT function, recognition chemistry, transduction mechanism, sample conditioning, and matrix composition must be considered together when determining whether a reported signal can be attributed to the intended recognition event.

### 1.4. Design Framework

The design framework developed here connects material and device reproducibility with recognition-layer accessibility, sample transport, and matrix-matched validation. Relevant choices include CNT type, semiconducting fraction or chirality where appropriate, dispersion chemistry, network morphology, contact geometry, and fabrication process. These choices should therefore be evaluated through distributions across devices and batches, rather than through a single representative response. The commercialization framework discussed by Li et al. provides a broader context for connecting device fabrication to packaging and scale-up, without constituting evidence that those steps have already been completed for a particular biosensor [5].

The second layer determines whether the intended molecular interaction is accessible, selective, and coupled to the chosen transduction mechanism. Receptor orientation, attachment chemistry, and surface coverage must be considered together with nonspecific adsorption. Electrical coupling can be weakened when the relevant charge redistribution occurs too far from the channel, while a protective coating can also limit analyte access. A synthetic optical recognition corona has a different purpose from a coating designed primarily to suppress fouling, and each function requires its own experimental support [6,24,25]. The design objective is therefore not simply maximal binding affinity or maximal passivation, but a functional interface under the intended measurement conditions.

The third layer controls what reaches that interface and when. Separation, enrichment, dilution, delivery, and washing can be used to manage the sample presented to the sensor. CNT-enabled plasma extraction and capture followed by Raman identification illustrate distinct ways of incorporating nanostructures into this part of the workflow [27,38]. Their significance lies in the functions actually demonstrated, rather than in an assumed universal integrated platform. A proposed modular chain from raw sample to conditioned sample and controlled readout should make every processing step explicit, because pretreatment changes both the matrix and the interpretation of reported performance. These design layers affect different analytical outcomes. Receptor geometry primarily affects target accessibility and transduction coupling; surface engineering affects nonspecific adsorption, baseline stability, and receptor retention; and sample conditioning affects target recovery, matrix background, and delivery to the sensing region. An improvement in one layer therefore cannot be assumed to improve the complete assay unless the other layers remain compatible.

## 2. Interfacial Physics in Complex Biological Media

### 2.1. Electrostatic Gating and Debye Screening

Within the dilute-electrolyte description, the Debye screening length decreases as ionic strength increases. This length is a characteristic electrostatic decay scale rather than a sharp boundary separating detectable and undetectable molecules. It should be distinguished from the plane of electron transfer, which refers to the spatial region where electron-transfer processes occur at an electrochemical interface. The Debye screening length describes attenuation of electrostatic perturbations in solution and does not itself define the plane of electron transfer [39]. Its relationship to receptor size, linker length, and binding geometry is especially important when a large protein complex extends away from the CNT surface. Charge located far from the channel can be strongly screened even when the receptor has high affinity for the target. Experiments on point-functionalized CNT transistors by Sorgenfrei et al. directly connected the amplitude of single-molecule electrical signals to ionic screening and charge proximity, supporting the need to consider the location of the transducing interaction [32].

**Figure 2:**
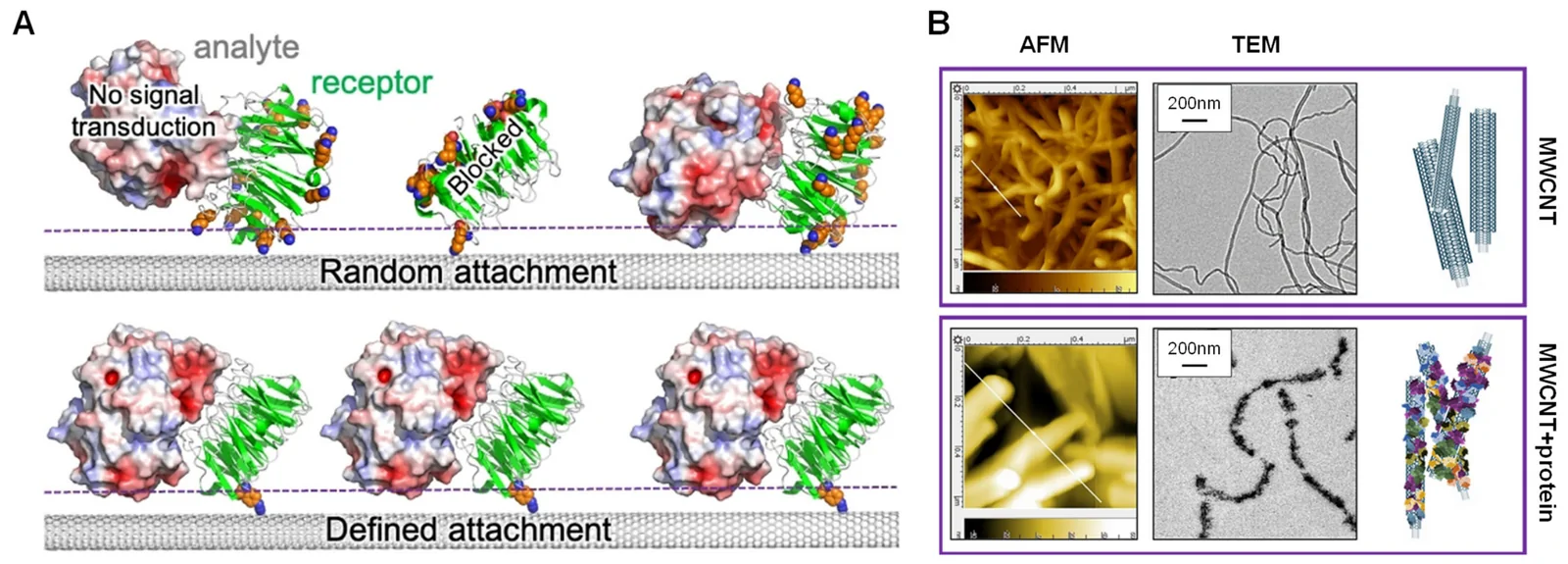
Receptor geometry and biological coating formation at CNT interfaces. (**A**) Random and defined protein attachment geometries illustrating how receptor presentation affects ligand access and proximity within the effective Debye screening region. (**B**) Schematic, transmission electron microscopy (TEM), and atomic force microscopy (AFM) comparisons of multiwalled carbon nanotubes before and after spontaneous serum-protein adsorption. The protein layer represents an uncontrolled biological corona and should not be interpreted as an intentionally immobilized receptor layer. (**A**) Reproduced from Xu et al. [24] under the Creative Commons Attribution License. (**B**) Adapted from García-Hevia et al. [25] under CC BY 4.0; orientation and labels modified.

The resulting dependence on geometry is illustrated in Figure 2A, which relates receptor attachment to the position of the target-induced electrostatic perturbation. The local molecular layer matters alongside bulk ionic strength because it determines which charged regions can approach the nanotube. A schematic screening distance is therefore a guide to coupling, not a universal detection boundary.

### 2.2. Orientation of Recognition Elements

Immobilization determines which part of a recognition element faces the nanotube and which part remains exposed to the solution. Common strategies include noncovalent adsorption, covalent attachment, affinity-based immobilization, entrapment, and immobilization within polymeric or hydrogel matrices [40]. Randomly attached proteins can retain target affinity while presenting heterogeneous binding geometries. Consequently, the number of bound targets and the magnitude of the electrical response need not vary proportionally. The recognition event must produce a charge redistribution or another perturbation that couples to the active conduction pathway. Orientation is thus an electrical design variable as well as a biochemical one [24].

Xu et al. tested this principle using β-lactamase inhibitory protein 2 (BLIP2) as a receptor for the β-lactamase TEM-1 [24]. They introduced p-azido-L-phenylalanine at defined protein positions and used pyrene-based attachment chemistry to control receptor orientation on semiconducting CNTs. TEM-1 addition increased conductance for the residue-41 attachment configuration but decreased conductance for the residue-213 configuration. The authors related the opposite responses to the different local charge patches presented near the channel. Figure 2A captures the underlying geometric argument: changing the tethering site changes the surface sensed by the CNT, even though the recognized protein remains the same. The study therefore connects a molecular design variable directly to signal polarity and supports optimization of receptor presentation alongside affinity.

Receptor orientation should be evaluated using complementary chemical, structural, and functional measurements. Site-specific labeling or coupling can confirm whether the intended residue or domain has been used for immobilization, but it does not by itself demonstrate that the binding site is exposed or that all receptors adopt a productive orientation. Surface-sensitive methods, including X-ray photoelectron spectroscopy, fluorescence labeling, quartz-crystal microbalance, and ellipsometry, can provide information on surface modification, receptor loading, and layer thickness. These measurements should be combined with an accessibility assay, such as binding of a labeled target or an epitope-specific probe, because total receptor loading does not necessarily indicate functional accessibility.

For CNT-based electrical sensors, orientation should ultimately be evaluated through a controlled functional comparison. Randomly immobilized and site-directed receptors should be compared using the same CNT architecture, receptor loading, target concentration, ionic strength, incubation time, and measurement protocol. Relevant performance metrics include the target-induced conductance or current change, response polarity, calibration slope, limit of detection, dynamic range, response time, nonspecific response, and device-to-device variation. Xu et al. demonstrated the mechanistic importance of receptor position by attaching BLIP2 to semiconducting CNTs at defined residues. Attachment at residues 41 and 213 produced different conductance responses to TEM-1, including opposite response polarity, despite using the same receptor–target pair [24]. This result supports the role of receptor position and local charge geometry, but it should not be interpreted as a universal quantitative sensitivity improvement over random immobilization.

This comparison also explains why random immobilization can yield heterogeneous responses. Different receptors on the same surface may present productive, weakly coupled, or inaccessible binding configurations. Their electrical contributions need not reinforce one another. Measurements of binding activity and surface loading are consequently necessary but insufficient to establish a uniform electrical interface. The useful design variable is the distribution of recognition geometries that remains after immobilization, as well as the amount of receptor attached.

Receptor dimensions provide an additional route for controlling electrostatic coupling. Kim et al. used antibody-binding fragments on CNT-FETs specifically to reduce the distance between the charged target and the nanotube [41], while earlier prostate-specific antigen–α1-antichymotrypsin (PSA-ACT) work tuned linker-to-spacer ratios to improve access within the effective screening region [42]. These studies complement the orientation control of Xu et al. [24]: receptor engineering can change not only affinity and surface coverage, but also the distance and geometry through which binding is electrically communicated. Work on aptamer-FETs outside the CNT platform further showed that target-induced conformational changes of compact aptamers can remain detectable at physiological ionic strength, providing a useful mechanistic comparison for CNT receptor design [43].

### 2.3. Ionic Strength, pH, and Charge State

Ionic strength and pH influence both the electrical environment and the recognition reaction. Changing salt concentration modifies electrostatic screening, while changing pH alters the protonation states of the analyte, receptor, and surface groups. A current change observed after exchanging solutions can therefore contain a substantial background contribution unrelated to specific binding. Comparisons should hold the measurement solution constant or explicitly separate the response to the solution change from the response to the target [32,33].

A comparison with earlier CNT-FET studies shows that this problem has been approached through several distinct design strategies rather than a single universal solution. So et al. used a thrombin-binding aptamer on SWCNT-FETs, and Maehashi et al. later compared aptamer- and antibody-modified CNT-FETs for immunoglobulin E (IgE), illustrating how a smaller recognition element can reduce the separation between the target-associated charge and the nanotube [10,11]. Kim et al. similarly emphasized the advantage of an aptamer over a full antibody in an electrolyte environment resembling serum [12]. By contrast, Kulkarni and Zhong operated a SWCNT-FET at high frequency to detect beyond the conventional dc Debye-screening limit [44]. The aptamer-based studies primarily addressed the distance between the target-associated charge and the CNT surface, whereas the high-frequency device addressed the electrical measurement regime in which ionic screening limits the response. These studies therefore address different mechanisms: compact recognition elements reduce the distance between the target-associated charge and the CNT, high-frequency operation changes the electrical measurement regime, and interface engineering aims to reduce drift and preserve signal stability. Their results should not be interpreted as directly comparable improvements in intrinsic sensitivity.

Engineering the local ionic environment offers another approach. Albarghouthi et al. combined a poly(oligo(ethylene glycol) methyl ether methacrylate) (POEGMA) interface with antibody printing and a measurement protocol designed to reduce drift in CNT transistors. Their operation in 1× phosphate-buffered saline (PBS), rather than a diluted PBS formulation, illustrates an attempt to address screening and stability together [45]. Physiologically relevant ionic strength, however, is distinct from the full compositional complexity of a biofluid. For a new coating, the appropriate next comparison is therefore not only against a low-salt control, but also against the intended matrix under the same capture and readout workflow.

### 2.4. Transduction Mechanisms Beyond Simple Field Effect

CNT transduction depends on both device architecture and interface design. For electrochemical CNT electrodes, an enhanced current does not necessarily prove that CNT sidewalls are intrinsically electrocatalytic. In porous CNT layers, the response may also contain contributions from thin-layer diffusion, mass transport within the pores, increased electroactive area, and electron-transfer kinetics at exposed CNT ends or other active regions. Single-entity measurements and transport-aware modelling can help distinguish these contributions from a genuine change in heterogeneous electron-transfer kinetics. This distinction is important when transferring conclusions from porous electrochemical electrodes to CNT-FET or optical biosensor architectures [1,46]. Studies of nucleic-acid and protein sensors illustrate this dependence across different channel geometries, surface chemistries, and recognition formats [41,42,47,48,49,50]. In CNT-network devices, the measured response can arise from several electrically distinct locations. Binding near the nanotube channel may modulate local carrier density or scattering, whereas binding near a metal–CNT contact may alter charge injection. Target capture can also change the resistance of tube–tube junctions or the percolation pathway through the film. A concentration-dependent current change alone cannot identify which contribution dominates without controls based on contact geometry, channel length, recognition-site location, or device architecture [13,14,15,16].

Mechanistic studies of nucleic-acid sensing reinforce this point. Gui et al. used CNT-network FETs with different metal contacts and location-selective capping to show that DNA-hybridization responses could be dominated by modulation of the metal–SWCNT junction rather than by uniform channel gating [13]. Sorgenfrei et al. subsequently resolved single-molecule DNA-hybridization kinetics using point-functionalized CNT-FETs [14], while suspended-CNT and channel-length studies showed that device geometry can change the magnitude and electrical signature of hybridization [15,16]. Consequently, a concentration-dependent response should not be interpreted independently of contact design, channel geometry, and the location of the recognition chemistry.

CNT-modified electrochemical electrodes constitute a separate class. Their current or impedance can reflect processes at the electrode–electrolyte interface, rather than field-effect modulation of a semiconducting nanotube channel. The phage-functionalized gold nanoparticle/tungsten trioxide/multiwalled carbon nanotube (AuNP/WO_3_/MWCNT) electrode of Abdelhamied et al., for example, is an impedimetric pathogen sensor and should not be treated as a two-terminal CNT chemiresistor or a CNT-FET [23]. Likewise, optical emission changes require their own photophysical interpretation. Maintaining these distinctions prevents a design rule derived for one mechanism from being applied indiscriminately to another.

### 2.5. Integrated Recognition–Transduction Design

Recognition chemistry and device physics should be designed together. Affinity determines whether binding is favorable, but the resulting signal depends on where the interaction occurs and how it perturbs the selected transducer. For electrostatically coupled devices, receptor orientation, attachment distance, electrolyte composition, and the location of the active channel must be considered as an interdependent set. For other electrical architectures, the relevant contacts, junctions, or electrochemical interfaces must instead be identified [23,24].

A useful development sequence is to establish recognition under the intended chemical conditions, identify a plausible transduction mechanism, and then test whether that mechanism remains informative after exposure to the target matrix. In practice, the interface should produce a reproducible signal when the target is present while remaining stable when the same sample matrix is measured without the target. Maximizing affinity or buffer response alone cannot satisfy this criterion. That interface must also remain functional as the biological coating evolves during sample exposure.

Table 1 summarizes matrix-dependent limitations, their consequences for CNT sensing, and corresponding surface-engineering and sample-conditioning strategies, together with representative evidence.

## 3. Biofouling and Surface Engineering

### 3.1. Formation of the CNT Protein Corona

The interface that contacts a biological sample is not necessarily the interface produced during sensor fabrication. Proteins and other sample components can adsorb onto accessible nanotube surfaces, creating a biological coating whose composition depends on both the material and its environment. This protein corona changes which molecular groups remain exposed and can modify access to immobilized receptors. Surface chemistry must therefore be understood as an evolving property during sample exposure, rather than solely as a fabrication specification [25].

García-Hevia et al. investigated spontaneous CNT biocorona formation by combining electron and atomic force microscopy with biochemical characterization [25]. The comparison of MWCNTs before and after protein exposure revealed a change in nanotube coating and apparent dimensions, providing the direct structural evidence represented in Figure 2B. Protein profiles also varied with the nanotube and exposure conditions, and sera from different human donors produced different profiles on the same nanotube type. These observations show that a surface specification established before exposure does not fully determine the interface encountered during measurement. The coating observed in Figure 2B represents spontaneous protein adsorption onto the CNT surface rather than deliberate receptor immobilization. Proteins from the surrounding serum can approach and attach to exposed CNT surfaces through noncovalent interactions, forming a protein-rich layer around the nanotube. This layer can partially cover the CNT surface and alter the accessibility of subsequently immobilized or exposed recognition sites. The microscopy shows changes in the apparent CNT coating after serum exposure, whereas the donor comparison indicates that the protein composition associated with the CNT surface differs among serum samples from different individuals. These observations demonstrate interface variability, but neither observation alone quantifies the resulting electrical sensing error. Here, “donor” refers to the individual from whom the serum sample was obtained, and “variability in composition” refers to differences in the proteins associated with the CNT surface after exposure to serum from different individuals.

### 3.2. Designed Recognition Coronas vs. Uncontrolled Fouling

An intentionally designed corona must be distinguished from uncontrolled fouling. In the protein-recognition system reported by Bisker et al., a phospholipid–polyethylene glycol (PEG) corona is part of the selective optical sensor rather than an unwanted surface contaminant [6]. The relevant distinction is functional: a coating should support the intended recognition and transduction while limiting competing interactions. Neither the presence of protein on a CNT nor a high degree of polymer coverage can establish this outcome without appropriate controls. The spontaneous coating formation shown in Figure 2B therefore differs from the purposeful recognition interface discussed here.

### 3.3. Antifouling Strategies

Antifouling strategies seek to reduce unwanted adsorption without removing access to the recognition sites. Hydrophilic polymers, PEG and PEG–lipid coatings, zwitterionic layers, protein-based blocking, mixed surface layers, and selective functionalization offer different ways of pursuing this balance. They should be selected according to the nanotube attachment chemistry, transduction mechanism, and sample environment. A coating that performs well as a colloidal stabilizer need not provide the optimal interface for a surface-bound electrical sensor [6,25].

Earlier CNT-FET studies also show that passivation cannot be separated from receptor presentation. Cid et al. used Tween 20 to suppress nonspecific adsorption in an immunoglobulin G (IgG) CNT-FET, whereas a related physiological-condition format used polyethylenimine (PEI), glutaraldehyde coupling, and glycine blocking [47,49]. Mao et al. introduced Au nanoparticle-antibody conjugates on CNT channels and verified low response to mismatched proteins [48]. Together with modern POEGMA interfaces [45], these studies show an evolution from empirical blocking toward engineered interfacial layers whose adsorption resistance, receptor accessibility, and electrical stability are evaluated together.

The need for this balance was recognized early in CNT biointerfaces. Chen et al. demonstrated that nonspecific protein adsorption is extensive on pristine SWCNTs and used noncovalent polymer functionalization to suppress this adsorption while retaining specific recognition [56]. More recent device studies separate passivation of the electrical structure from passivation of the biological interface. Chang et al. placed a graphene-oxide membrane above SWCNT channels to preserve nanotube electronic properties while providing attachment sites for biorecognition [57], and Albarghouthi et al. systematically evaluated photoresist, dielectric, and PEG-containing passivation strategies for solution-gated CNT devices [31]. Together with the POEGMA-based biomarker assay discussed above [45], these results show that antifouling is most effective when leakage control, receptor immobilization, and resistance to nonspecific adsorption are treated as coupled but experimentally separable functions.

PEG-containing layers are particularly instructive because they can serve different roles. A PEG–lipid corona can support molecular recognition in an optical SWCNT system [6], whereas a PEG-like polymer brush can be incorporated into an electrical assay to address nonspecific adsorption and the ionic environment [45]. These outcomes do not make all PEG-containing coatings interchangeable. Chain presentation, attachment stability, and the location of the receptor relative to the transducer remain relevant. The identity of the polymer is therefore only the starting point for evaluating a functional interface.

Zwitterionic coatings offer another route based on highly hydrated surface chemistry. Ladd et al. compared polymer and self-assembled-monolayer surfaces exposed to human serum and plasma and found strong resistance to nonspecific adsorption for zwitterionic polymer brushes [52]. This is supporting surface-science evidence, rather than direct validation of a CNT biosensor. Transfer to CNT devices requires confirmation that the coating can be attached reproducibly, accommodate recognition elements, and preserve the selected electrical or optical response.

Evidence from broader electrochemical interfaces also illustrates how aggressive matrix protection can be achieved without completely isolating the transducer. Sabaté del Río et al. reported a porous cross-linked bovine serum albumin (BSA)/nanomaterial coating that retained affinity-based electrochemical sensing after prolonged plasma exposure, including formulations containing CNT networks [53]. Sato and Konno used an amphiphilic phospholipid polymer to disperse and immobilize MWCNTs while providing antifouling behavior at an electrode [58]. These systems are not direct substitutes for CNT-FET coatings, but they demonstrate a general principle for biointerface design: a hydrated or protein-rich barrier can be engineered to preserve analyte transport while reducing uncontrolled adsorption. Translation to a CNT transistor still requires verification that the added layer does not move the recognition event outside the electrically useful region.

Protein-based passivation can preoccupy adsorption sites, although the resulting coating must remain functional during competitive exposure. García-Hevia et al. addressed this issue by engineering recombinant proteins with a CNT-binding component to create a more controlled surface coating [25]. Their intervention reduced unwanted association with environmental proteins and provided experimental support for controlled protein-based passivation. In a biosensor, the corresponding test is whether that protection preserves access to the intended recognition sites and maintains target-dependent transduction. A passivation layer can reduce nonspecific adsorption by limiting access of background proteins, but it can also reduce target transport or increase the distance between the target and the electrically or optically sensitive CNT interface. Its benefit should therefore be assessed from the target response, background response, response drift, and retained receptor accessibility measured together. Conventional bovine serum albumin blocking pursues a related surface-protection objective, but its performance cannot be assumed from the presence of a preadsorbed protein layer alone.

### 3.4. Trade-Off Between Antifouling and Analyte Access

Increasing coverage may reduce access by unwanted adsorbates, but the same physical barrier can impede the target. A dense coating can introduce additional transport resistance, constrain receptor motion, or move a binding event farther from the electrically sensitive region. For electrostatically gated CNTs, this last effect couples antifouling design directly to receptor geometry and ionic screening [24]. The appropriate design target is therefore the net analytical benefit after sample exposure, rather than the lowest adsorption signal measured in isolation.

These properties are most informative when assessed together. An initial target calibration characterizes the fresh interface; a matrix-only exposure reveals background evolution; and a subsequent target challenge tests retained activity. Differences in recovery, response time, drift, and calibration shape then provide a more informative basis for choosing a coating than either adsorption suppression or buffer sensitivity alone. This approach also identifies interfaces that remain apparently clean but have lost useful recognition capacity.

A target-dependent change in electrical current or fluorescence should not be interpreted as evidence of specific recognition without appropriate controls. A minimum control set should include: (i) a matrix-only control without the target to measure nonspecific adsorption and background drift; (ii) a non-target or mismatched analyte control to test molecular selectivity; (iii) a receptor-free or nonbinding-receptor control to determine whether the signal originates from the recognition element; (iv) a buffer-exchange or ionic-strength control to separate solution-induced electrostatic changes from target binding; and (v) a pH control performed at matched ionic strength because pH can alter receptor charge, surface chemistry, and CNT fluorescence. For complex biological samples, the response should also be tested after exposure to the relevant matrix alone and after recovery or spiking of the target into that matrix. Specific recognition is most convincingly supported when the target response is retained in the matrix, is absent or substantially reduced in the non-target and receptor-free controls, and remains distinguishable from the response caused by ionic-strength or pH changes.

The appropriate interpretation also depends on the transduction mode. In electrical CNT sensors, current changes can arise from electrostatic gating, contact or junction effects, ionic redistribution, adsorption-induced mobility changes, or baseline drift. In optical SWCNT sensors, fluorescence changes can result from corona rearrangement, local dielectric changes, quenching, nanotube aggregation, or optical-background effects in addition to selective analyte recognition [7,21,59,60,61]. Accordingly, selectivity should be demonstrated using target-versus-nontarget controls and matrix-matched controls, while specificity should be supported by receptor-dependent measurements and, where possible, an orthogonal confirmation of target binding.

### 3.5. Reporting Recommendation

Reports of antifouling performance should specify the matrix, dilution, exposure duration, temperature, and sequence of incubation and washing. Serum and plasma should be identified separately, and pooled samples should not substitute silently for individual-donor testing. Where a study uses a purified protein challenge, its conclusions should remain limited to that challenge rather than imply resistance to the entire biofluid.

Finally, the number of independent devices, coating preparations, and biological samples should accompany averaged responses. Repeated readings from one interface characterize instrumental repeatability, whereas separately prepared interfaces probe fabrication and functionalization reproducibility. Reporting both makes it possible to judge whether an antifouling strategy can support the next stage of development: controlled sample delivery and integration into a complete assay [5].

## 4. System-Level Integration from Sample to CNT Sensor

### 4.1. Why Fluidics Matters

A rapid response of the CNT transducer does not imply a rapid assay. Before a measurable interaction can occur, the target must reach an accessible recognition site in sufficient quantity. In surface-based biosensors, diffusion, convective delivery, and binding kinetics jointly determine this process. Squires et al. showed why transport can be as important as the surface reaction when interpreting response times and designing measurements [28]. CNT sensitivity therefore needs to be evaluated within the geometry and flow conditions that deliver the sample, rather than treated as an isolated material property. Diffusion determines how rapidly the target reaches the sensing surface when mixing is limited, whereas convection determines the rate at which fresh sample is delivered under flow. Increasing the flow rate can improve target delivery but can also reduce residence time and limit binding. Channel height, width, length, surface-to-volume ratio, and the position of the CNT sensing region therefore affect both response time and capture efficiency. The measured response is consequently determined by the combined transport and binding process rather than by the CNT transducer alone [17,28,29].

In an integrated CNT biosensor, the observed response time is determined by the combined effects of diffusion, convection, target–receptor binding, and signal transduction. Binding kinetics provide a second limitation. Even when transport to the CNT surface is rapid, a slow association rate can delay signal development, whereas a slow dissociation rate can prolong washing and recovery. The measured signal is consequently determined by the interplay between transport and reaction rather than by the intrinsic CNT transducer alone. In integrated platforms, flow rate and channel geometry should therefore be reported together with receptor density, incubation time, washing conditions, and the target concentration. Microfluidic CNT biosensor studies illustrate how focusing, channel design, and controlled delivery can increase the amount of target presented to the sensing region, but the resulting response should be interpreted as a property of the integrated assay rather than of the CNT material alone [17,28,29].

**Figure 3:**
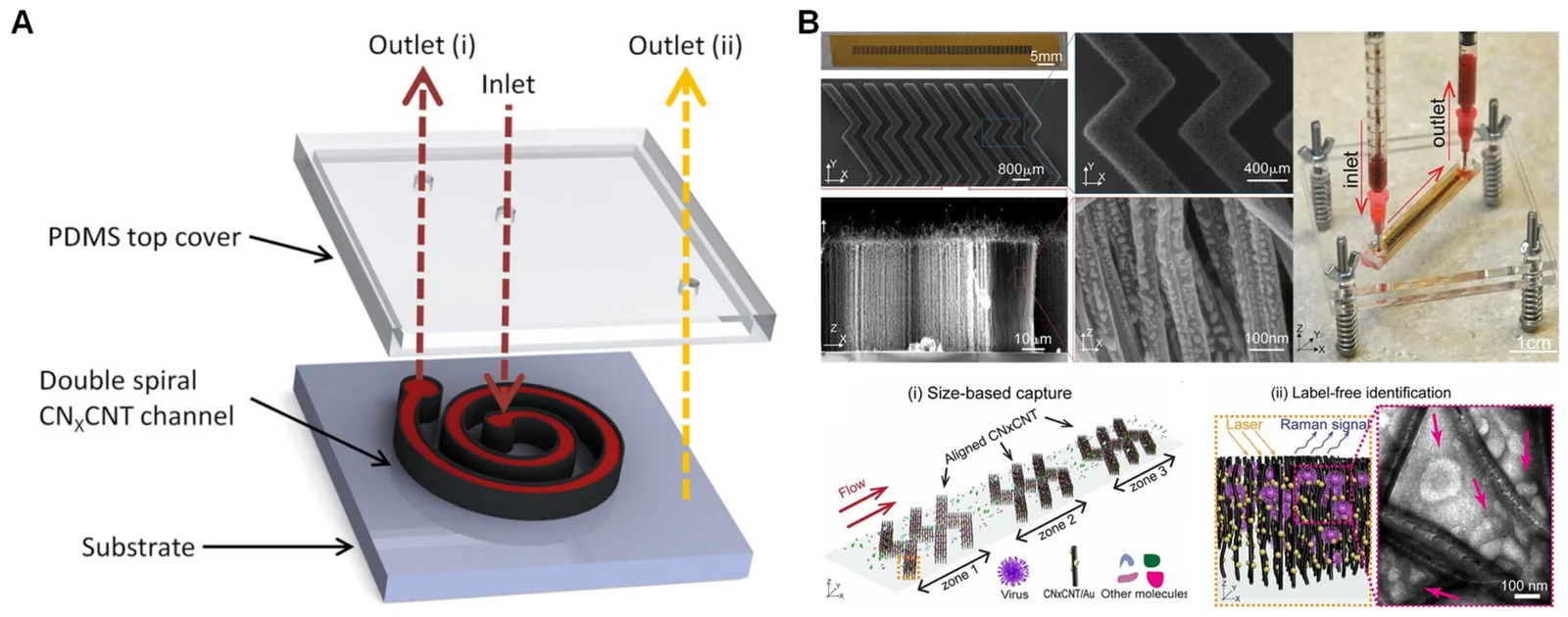
CNT-enabled sample preparation and virus capture. (**A**) Assembly of a CNT-wall microfluidic device for plasma extraction, with separate outlets for blood flow and plasma collection. (**B**) The VIRRION platform, showing patterned CNT arrays, the assembled device, and size-based virus capture coupled to gold-assisted Raman identification. A and B represent separate experimental platforms. (**A**) Reproduced from Yeh et al. [27] under CC BY 4.0. (**B**) Adapted from Yeh et al. [38] under CC BY 4.0; selected components rearranged.

The practical implication is that transport belongs in the sensor specification. Sample volume, channel geometry, flow rate, incubation mode, and wash conditions should accompany a reported calibration. When these quantities change, a different assay may have been created even if the CNT device and receptor remain identical. Figure 3 places this issue at the center of the workflow by showing preparation and capture as explicit functions upstream of readout [27,38].

### 4.2. Microfluidic Delivery and Washing

Integration studies show that fluidics can do more than simply deliver a fixed sample volume. Han and Jang combined electrohydrodynamic focusing and dielectrophoretic concentration with a SWCNT-FET immunosensor to increase the number of flowing bacteria presented to the sensing region [29]. Vlandas et al. integrated a boronic-acid-functionalized SWCNT liquid-gated transistor into a microfluidic channel for affinity-based sugar sensing and used the platform to distinguish charge-scattering and surface-charge contributions under different coupling chemistries [17]. These examples complement the separation-focused devices in Figure 3: microfluidics can define transport, enrich targets, and provide a controlled environment for mechanistic interrogation, but each added operation changes the sample history that must be reported with the calibration.

Ashiba et al. investigated a complementary optical integration in which CNT fluorescence labels were measured using a microfluidic V-trench configuration with surface-plasmon-assisted excitation [62]. The arrangement brought sample delivery and optical interrogation into the same sensor chip and demonstrated measurement of the CNT-label fluorescence. This study illustrates how sample delivery and optical interrogation can be co-designed. The proposed analyte detectability was modeled, so this experiment does not establish measured pathogen performance. It nevertheless shows how an optical readout imposes constraints on the chip beyond those required for liquid transport alone.

### 4.3. Preconcentration and Separation

More recent integration work brings this logic closer to whole-blood analysis. A 2026 pressure-driven platform combined dimensionally confined gravitational plasma separation with downstream CNT-FET detection of carbohydrate antigen 19-9 (CA19-9), reporting 84.9% plasma yield and 99.9% blood-cell removal before sensing [30]. Earlier, Li et al. coupled CNT multilayer capture with microfluidic loop-mediated isothermal amplification (LAMP) for *E. coli* O157:H7 identification [63]. Although the first platform uses CNT-FET transduction and the second couples CNT-assisted capture to LAMP, both show why sample conditioning, target recovery, and downstream detection should be evaluated as linked operations. These studies should not be interpreted as equivalent demonstrations of CNT biosensing. In the plasma-extraction device, the CNT structure performs a separation function and albumin recovery is measured by a separate downstream assay. In the latter platform, plasma separation precedes CNT-FET detection. The distinction is important because the first study evaluates sample preparation, whereas the second evaluates an integrated preparation-and-detection workflow.

Conditioning can reduce the complexity of the material reaching the sensor or increase the amount of target available for capture. Candidate operations include magnetic capture, membrane filtration, size-based separation, deterministic or inertial separation where appropriate to particle dimensions, affinity enrichment, and on-chip dilution. Their purpose must be stated precisely. Removal of cells from blood produces a different sample fraction; selective capture enriches a particular target population; dilution reduces both matrix components and analyte concentration. These interventions should be evaluated as transformations of the original sample, with recovery and selectivity assessed at each relevant step.

Dilution, separation, and enrichment affect the sample presented to the CNT sensor in different ways. Dilution can decrease the concentration of both the target and interfering matrix components. Its benefit therefore depends on whether the reduction in background improves the signal-to-background ratio sufficiently to compensate for the loss in target concentration. Separation can remove cells or other interfering components, but target loss may occur through incomplete recovery, adsorption to the separation material, or partitioning into the discarded fraction. Enrichment can increase the target concentration in the processed sample, but the resulting analytical benefit depends on the recovery, enrichment factor, processing volume, and additional background introduced during the procedure.

These effects should be evaluated using the complete sample-to-answer workflow. The concentration delivered to the CNT sensor should be distinguished from the concentration in the original specimen. Accordingly, studies should report the initial and post-conditioning target concentrations, target recovery, dilution or enrichment factor, processed sample volume, processing time, matrix background, and detection limit. CNT-enabled plasma extraction, microfluidic focusing, and CNT-assisted capture illustrate how sample conditioning can alter recovery and the sample delivered to the downstream sensor [27,29,30,63]. A lower detection limit measured after conditioning should therefore be referred both to the processed sample and, where appropriate, to the original sample before conditioning.

Yeh et al. developed a plasma-extraction microdevice in which aligned nitrogen-doped CNTs formed porous walls around a double-spiral flow channel [27]. Whole blood flowed along the channel while plasma passed through the CNT walls into a separate collection path, as illustrated in Figure 3A. The authors reported approximately 80% albumin recovery, assessed using a separate downstream assay. The result demonstrates that a CNT structure can contribute to sample preparation while retaining a measurable fraction of a soluble biomarker. It does not make the extraction chip an electrical albumin sensor. This separation of preparation and detection is important because the recovery achieved upstream constrains the amount of target available to any subsequent transducer.

Yeh et al. subsequently developed virus capture with rapid Raman spectroscopy detection and identification (VIRRION), which combined size-based virus capture with gold-assisted Raman identification [38]. In the architecture summarized in Figure 3B, the CNT array supplies a capture structure, while the gold-assisted Raman measurement provides spectral information for identification. The study joins capture and readout more closely than the plasma-extraction device, but the two functions remain experimentally distinguishable. Its contribution is the integration of nanostructured capture with an optical identification step, rather than field-effect detection or fluorescence modulation by a SWCNT recognition corona. Together, the two Yeh studies show why the function performed by the nanotube must be specified at every stage of a sample-processing workflow.

### 4.4. Multiplexing and Sensor Arrays

Electrical integration has also progressed beyond single isolated channels. Lee et al. integrated 64 CNT-based sensing elements with complementary metal–oxide–semiconductor (CMOS) signal-processing circuitry on one chip for enzymatic glutamate detection [64]. Xu et al. later demonstrated reconfigurable multiplexed devices in which distinct DNA-wrapped SWCNTs were positioned on prepatterned electrodes and functionalized with different aptamers [65]. These studies illustrate two complementary scaling problems: electronic integration of many nominally similar channels and biochemical differentiation of channels for multiplex recognition. A clinically useful array requires both, together with fluidic uniformity and channel-specific calibration.

Fluidic multiplexing introduces additional questions about whether channels receive comparable samples and exposure histories. Unequal delivery or target depletion upstream can produce channel-dependent signals that resemble differences in recognition. Shared readout hardware also requires control of electrical or optical cross-talk. These system-level effects can be evaluated through reference channels, controlled sample distribution, and independent verification of the response assigned to each analyte, rather than through post hoc normalization alone.

### 4.5. Sample-to-Answer Architecture

The architectures in Figure 3A,B motivate a modular sample-to-answer workflow. Raw sample conditioning or enrichment precedes controlled delivery, after which CNT-enabled transduction and signal interpretation produce the analytical result. The modular sequence is a synthesis of the functions demonstrated across different studies, rather than a single instrument validated by those studies [27,38,62]. Its value is that each interface between modules can be tested for target loss, background changes, and compatibility with the next measurement step.

## 5. Electrical CNT Sensor Architectures for Pathogen and Biomarker Detection

### 5.1. Why Simplify the Transistor Architecture

**Figure 4:**
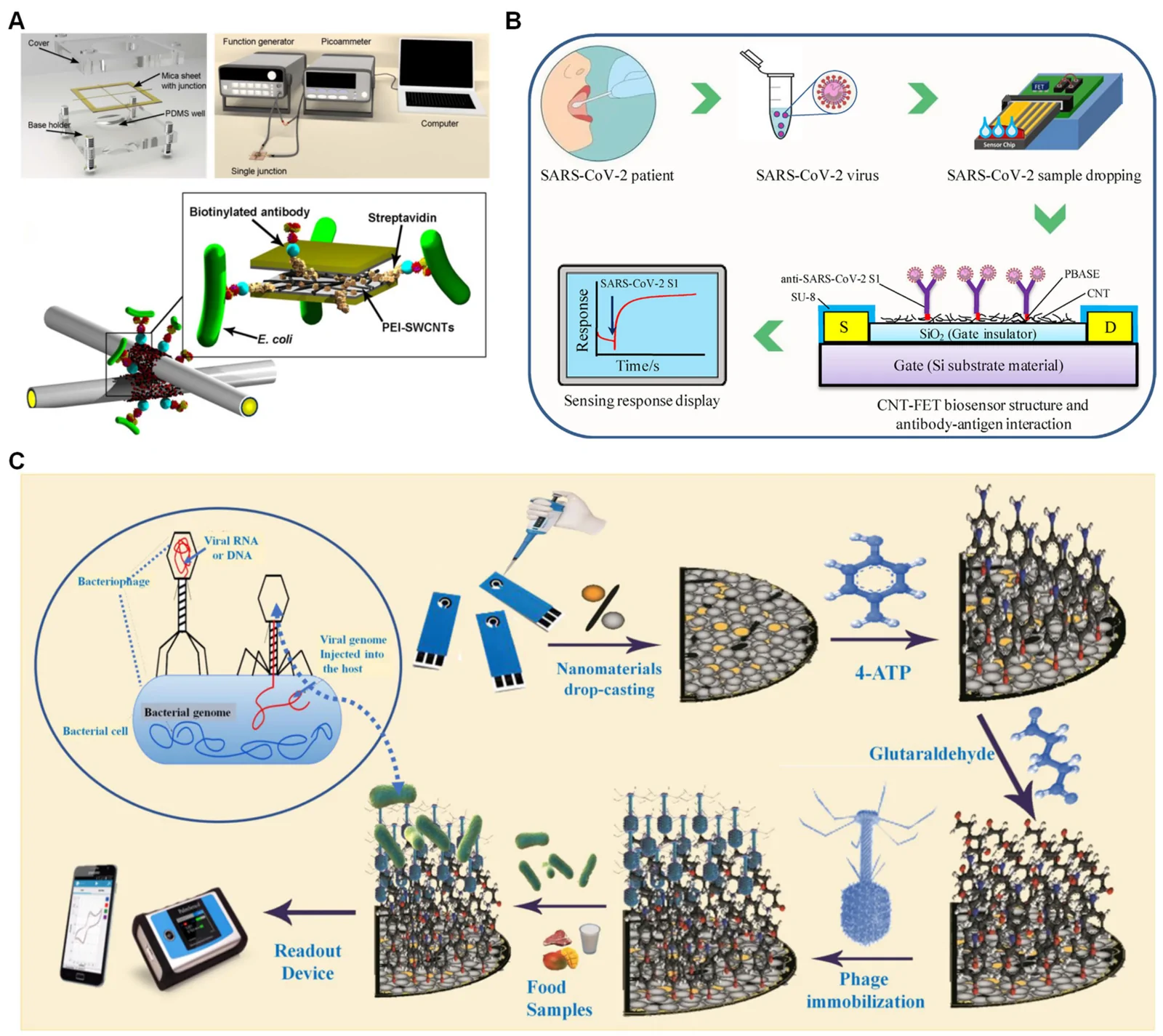
Electrical CNT-containing architectures for pathogen detection. (**A**) Crossed-wire SWCNT junction device, electrical measurement configuration, and bacterial capture concept. (**B**) Antibody-functionalized CNT field-effect transistor and SARS-CoV-2 S1 assay workflow. (**C**) Fabrication and operation of a phage-functionalized gold nanoparticle/tungsten trioxide/multiwalled carbon nanotube (AuNP/WO_3_/MWCNT) composite electrode for impedimetric detection of *Escherichia coli*. (**A**) Adapted from Yamada et al. [3] under the Creative Commons Attribution License; subpanels rearranged. (**B**) Reproduced from Zamzami et al. [26] under CC BY 4.0. (**C**) Reproduced from Abdelhamied et al. [23] under CC BY 4.0.

For disposable formats, simplification should be assessed at the cartridge level. Contacts must remain stable, sample-exposed regions must be controlled, and the reader must acquire a reproducible measurement from independently fabricated devices. A device that requires extensive individual characterization may transfer work from fabrication to calibration rather than reduce the total burden. These considerations make usability and manufacturability experimental questions, even when the underlying device geometry appears straightforward [4,5]. Figure 4 compares three pathogen-sensing architectures: a crossed-wire CNT junction (A), an antibody-functionalized CNT-FET (B), and a phage-functionalized AuNP/WO_3_/MWCNT composite electrode for impedimetric pathogen detection (C).

### 5.2. Junction, Chemiresistive, and Phage-Functionalized Composite-Electrode Sensors

In junction, chemiresistive, and phage-functionalized composite-electrode architectures, target-associated changes are monitored through an electrical conduction or interfacial impedance pathway. Depending on the device, the response can involve nanotube surfaces, tube–tube junctions, contacts, or the interface formed by the recognition layer. A concentration-dependent current change is useful analytically but does not by itself establish which of these contributions dominates. Mechanistic interpretation should remain tied to the actual device and its controls [33].

Yamada et al. developed a crossed-wire junction sensor by combining SWCNT-coated wires with antibody-mediated capture of *Escherichia coli* K-12 [3]. The architecture and sensing pathway are illustrated in Figure 4A. Current decreased with increasing bacterial concentration, and the authors reported detection down to 10^2^ colony-forming units (CFU) mL^−1^ within less than 5 min under their experimental conditions. The study illustrates how bacterial capture can be converted into a direct electrical signal without a conventional gated-transistor layout. The reported performance was evaluated using cultured *E. coli* K-12.

Earlier chemiresistive work provides useful context for the junction architecture. García-Aljaro et al. used aligned SWCNTs functionalized with antibodies to detect *E. coli* O157:H7 and bacteriophage T7 while challenging specificity with non-target microorganisms [66]. Mandal et al. used CNT thin films in a two-terminal format for whole M13 bacteriophage detection and explicitly compared the CNT device with microelectrodes lacking CNTs [67]. These studies, together with Yamada et al. [3], show that eliminating a gate electrode can simplify the measurement circuit, but the resulting response still depends on how adsorption, junctions, and contacts redistribute resistance through the CNT network. Adsorption can change the local charge environment or carrier scattering at the CNT surface, while bacterial capture can alter the electrical coupling between neighboring nanotubes or between the CNT film and the metal contact. In a network device, the measured resistance is therefore determined by the combined conductance of individual nanotubes, tube–tube junctions, and metal–CNT contacts. The relative contribution of these elements depends on network density, contact geometry, channel length, functionalization location, and the position of the captured target. A concentration-dependent resistance change alone cannot identify which contribution dominates without appropriate device controls.

Abdelhamied et al. used a different recognition and transduction scheme, immobilizing bacteriophages on an AuNP/WO_3_/MWCNT composite electrode for impedimetric pathogen detection in food samples [23]. Figure 4C relates the composite surface and phage recognition to the impedance readout. Here, the nanotubes form part of a modified electrochemical electrode, and the response is assessed through interfacial impedance rather than current through a crossed-wire junction. Comparison with Figure 4A therefore identifies a substantive architectural difference, even though both systems use CNT-containing interfaces for bacterial capture. Their analytical metrics must remain attached to their respective targets, sample matrices, and measurement procedures.

### 5.3. CNT-FET Sensing of Pathogens and Clinical Biomarkers

Zamzami et al. developed an antibody-functionalized CNT-FET for recombinant SARS-CoV-2 S1 recognition [26]. Their workflow couples capture at the CNT channel to an electrical transistor response, as summarized in Figure 4B. Figure 4B represents the specific antibody-functionalized CNT-FET workflow reported by Zamzami et al., rather than a generic immunosensor. The CNT channel serves as the electrically active sensing element, antibody functionalization provides recognition of recombinant SARS-CoV-2 S1, and the assay was evaluated using saliva fortified with the recombinant antigen. The figure should therefore be interpreted as an example of CNT-FET pathogen sensing under a defined sample condition, not as a general representation of all CNT immunosensors. They examined antigen-dependent response and tested saliva fortified with S1, establishing an experimental link between receptor functionalization and measurement in that matrix. The contribution is stronger than a buffer-only demonstration because the sample introduces additional background components. Its scope nevertheless remains antigen detection in fortified saliva; patient diagnosis requires evaluation of independently characterized clinical specimens.

Clinical-biomarker studies broaden the evidence beyond pathogen targets. Chen et al. combined wafer-uniform semiconducting CNT films with aptamers for amyloid-β40 (Aβ40) and amyloid-β42 (Aβ42) detection in full human serum, reporting sub-femtomolar response with device variation below 10% [51]. Earlier work detected amyloid-β in human serum using a carbon nanotube metal–semiconductor field-effect transistor (CNT-MESFET) architecture [68], while CNT-FET studies have addressed C-reactive protein (CRP) in fortified serum [69] and carcinoembryonic antigen (CEA) using an enlarged floating-gate interface [70]. These examples are more informative when compared by matrix, receptor, device variation, and validation design rather than by limit of detection (LOD) alone. A portable CNT-FET/clustered regularly interspaced short palindromic repeats (CRISPR)-Cas12a biochip for cardiac troponin I further illustrates how molecular amplification and electronic readout can be integrated when direct interfacial transduction is not the only design objective [71].

Additional applications include hemoglobin A1c (HbA1c)-related fructosyl valine sensing [18] and exosomal microRNA-21 (miRNA-21) detection in clinical plasma [72]. Across these studies, fortified matrices, prepared affinity targets, and patient specimens provide different levels of validation. Comparisons should therefore consider the receptor, matrix, sample preparation, and validation design alongside sensitivity.

### 5.4. Storage Stability and Batch Reproducibility

Storage stability and lot-to-lot variation are equally relevant to a disposable sensor. A device should be evaluated after the proposed storage procedure and then challenged with the target, rather than judged only by an unchanged baseline resistance. Similarly, repeated readings of one device should be distinguished from measurements on separately fabricated devices and independently functionalized lots.

### 5.5. End-to-End Assay Design Rule

For meaningful comparison, the complete procedure must remain in view. Cultured-bacteria junction sensing, recombinant S1 detection in fortified saliva, and impedance measurements in food matrices answer different analytical questions [3,23,26]. Their value is clarified by reporting the sample, target, recognition mechanism, readout, and validation level together. Simplification then becomes a disciplined design choice within a defined application, rather than a shortcut from a laboratory response to a claim of clinical readiness.

## 6. Optical SWCNT Biosensors in Biofluids, Cells, and Living Systems

### 6.1. NIR Fluorescence of Semiconducting SWCNTs

Near-infrared (NIR) fluorescence offers a complementary route for interrogating semiconducting single-walled carbon nanotubes (SWCNTs). Different nanotube chiralities have different optical transitions, allowing their emission to be resolved spectrally. Their photostability and emission in a biologically useful spectral region motivate measurements in environments where electrical contacts are difficult to implement. These properties make SWCNTs attractive optical probes, but they do not make every fluorescent nanotube a selective biosensor [8,9].

The optical sensing lineage predates many current CoPhMoRe implementations. Barone et al. demonstrated solution-phase SWCNT NIR sensing using glucose as a model analyte and established the utility of a photostable, environment-sensitive emission signal [59]. Satishkumar et al. showed reversible fluorescence quenching as a route to biomolecular sensing [60], and Heller et al. demonstrated multimodal optical signatures capable of differentiating several analytes and detecting responses inside living cells [61]. Kim et al. then engineered selective nitric oxide (NO)-responsive SWCNT fluorescence and demonstrated cellular and in vivo detection [73]. These studies established the central optical design principle used in later sections: molecular interactions must be translated into a reproducible change in nanotube emission rather than inferred from nanotube fluorescence alone.

The distinction between optical visibility and sensing is fundamental. An imaging probe needs to provide detectable emission at the location of interest, whereas a sensor additionally needs a reproducible relationship between an analyte interaction and the measured optical signal. Changes in emission intensity or wavelength can carry analytical information only when their origin is sufficiently constrained. A fluorescence change may reflect quenching, corona rearrangement, aggregation, or optical background in addition to selective analyte recognition. An optical response should therefore be assigned to target recognition only when it is supported by target, non-target, matrix, and concentration-dependent controls. Nanotube concentration, local optical conditions, and the molecular environment must therefore be considered alongside the intended recognition chemistry [6].

**Figure 5:**
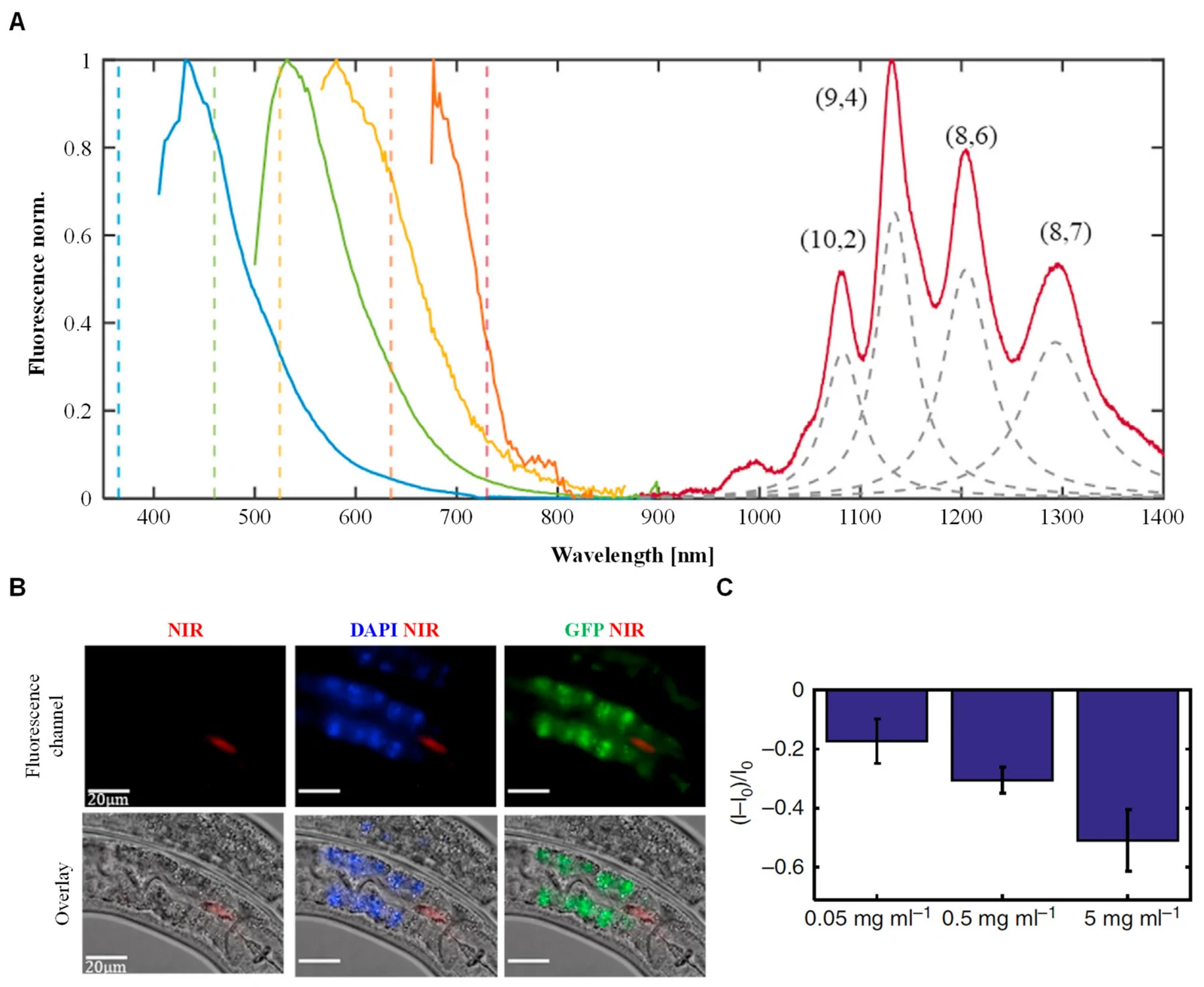
Optical access and analyte-responsive SWCNT fluorescence. (**A**) Normalized spectra illustrating the spectral separation between *Caenorhabditis elegans* autofluorescence and SWCNT NIR emission. (**B**) NIR fluorescence, overlaid with 4′,6-diamidino-2-phenylindole (DAPI) and green fluorescent protein (GFP) channels, and corresponding brightfield overlays showing SWCNT localization within the worms; scale bars, 20 μm. (**C**) Fibrinogen-dependent fluorescence response in 10% fetal bovine serum in phosphate-buffered saline (PBS). (**B**,**C**) show localization and analyte response in separate studies. (**A**,**B**) Adapted from Hendler-Neumark et al. [8] under CC BY 4.0; axis and channel labels reformatted. (**C**) Reproduced from Bisker et al. [21] under CC BY 4.0.

The normalized spectra in Figure 5A show the separation between *C. elegans* autofluorescence and DNA-functionalized SWCNT emission, illustrating the spectral rationale for NIR imaging [8]. Normalization permits comparison of spectral positions but does not establish relative absolute brightness. Figure 5B illustrates probe localization, discussed further in Section 6.3, whereas Figure 5C presents an analyte-dependent fluorescence response, discussed in Section 6.2.

### 6.2. Functionalization-Induced Molecular Recognition

The optical lineage also includes glucose sensing, which helped establish why NIR SWCNT emission is attractive for biological measurements. Barone, Parker, and Strano analyzed an SWCNT optical glucose-sensing concept in terms of tissue penetration, photostability, and sensor dynamics [74], and subsequent work developed SWCNT glucose-recognition chemistries toward continuous monitoring [75]. These studies predate CoPhMoRe but already frame the central optical problem: a stable fluorophore is useful only when the surrounding recognition chemistry converts analyte concentration into a reversible and calibratable spectral change.

Bisker et al. developed a phospholipid–PEG/SWCNT recognition phase and tested its response to fibrinogen in a serum-containing medium [6]. Figure 5C shows the concentration-dependent fluorescence response for fibrinogen added to 10% fetal bovine serum in phosphate-buffered saline. This experiment links the selected corona to analyte-responsive operation in the presence of additional biological components. Its significance differs from the localization evidence in Figure 5B: the measured variable changes with added target. At the same time, the defined, diluted serum condition remains distinct from undiluted human serum and from sensing inside a living organism.

The broader CoPhMoRe literature shows how adsorbed coronas can provide synthetic recognition sites for diverse analytes. Zhang et al. demonstrated nanotube-templated recognition for small molecules including riboflavin, L-thyroxine, and estradiol [19], and Kruss et al. screened polymer-wrapped SWCNTs for neurotransmitter responses, identifying reversible catecholamine-sensitive corona phases [20]. Subsequent work identified an insulin-responsive PEG-lipid/SWCNT construct that retained quantification capability in serum [21]. More recently, CoPhMoRe screening was adapted to SARS-CoV-2 proteins and tested in human-saliva conditions [76]. The progression from small molecules to proteins and biofluid operation demonstrates the breadth of corona-based recognition but also makes matrix-specific validation increasingly important because the recognition phase itself remains exposed to competing biomolecules.

Yoon et al. developed an enzyme-free uric acid recognition phase by screening DNA-wrapped SWCNTs and selecting an (AAT)10 corona [7]. They examined pH dependence, compared water and pooled-urine responses, and incorporated the sensor into an optical paper strip. The resulting fluorescence turn-on response connects recognition chemistry to a urine-oriented format. The urine protocol included ascorbate-oxidase pretreatment to reduce interference, so “enzyme-free” describes the uric acid recognition mechanism rather than every sample-handling operation. This distinction reinforces the need to evaluate optical recognition within its complete assay workflow.

### 6.3. Intracellular and In Vivo Operation

Optical interrogation is valuable where connecting electrodes to the sensing location or maintaining a liquid-gating configuration is impractical. It can provide spatial information as well as a time-dependent signal. The resulting evidence should nevertheless be separated into localization, imaging of biological motion or distribution, and analyte-responsive sensing. These levels require different controls, and success at one level does not establish the next [8,9].

In the *C. elegans* study, ingested DNA-functionalized SWCNTs were visualized mainly within the intestinal lumen [8]. The overlays in Figure 5B show probe localization, while time-dependent imaging revealed motion associated with the pharyngeal region. These observations demonstrate in-organism imaging of probe distribution and motion.

A separate line of work has moved from imaging feasibility toward chemically specific in vivo reporting. Iverson et al. used tissue-localizable SWCNT sensors to monitor nitric oxide associated with inflammation in mice and demonstrated long-term implantable sensing constructs [54]. Galassi et al. developed an optical SWCNT nanoreporter for endolysosomal lipid accumulation and applied it to disease and diet-related changes in hepatic macrophages in vivo [77]. These studies go beyond simple localization because the optical output is linked to a defined chemical or physicochemical change. At the same time, they underscore the calibration problem discussed above: local tissue environment, probe distribution, and analyte accessibility become inseparable from the measured signal.

Iizumi et al. investigated oxygen-doped CNTs as fluorescent labels and imaging probes, including mouse imaging after intravenous and oral administration [9]. The different administration routes produced different imaging contexts, while an immunoassay-label experiment provided a separate analytical demonstration. Taken together, these experiments support the use of engineered CNT emission across imaging and assay formats. They do not establish that anatomical fluorescence alone yields a quantitative map of an endogenous analyte.

Evidence of actual intracellular sensing requires an analyte-responsive experiment in addition to probe localization. Ulissi et al. used internalized DNA-wrapped SWCNTs to examine nitric oxide dynamics in A375 melanoma cells following treatment with an intracellular nitric-oxide-releasing compound [55]. Their analysis combined fluorescence changes with localization and a kinetic model. This provides a distinct example of intracellular analyte-related sensing, while also showing that the inferred signal depends on the occupied cellular compartments and the model used for interpretation.

### 6.4. Dynamic Sensing

The resistance of SWCNT fluorescence to conventional photobleaching makes repeated optical interrogation attractive for dynamic measurements. However, persistence of emission and persistence of sensing function are different properties. A probe can remain fluorescent while its recognition corona changes or its calibration drifts. Conversely, a decreasing signal may reflect analyte binding, motion, aggregation, or changes in optical collection rather than irreversible loss of the fluorophore. Dynamic measurements require a way to distinguish these alternatives.

Neurotransmitter sensing provides a stringent example of these requirements because both spatial and temporal information are important. Kruss et al. established reversible SWCNT responses to catecholamines [20], and subsequent NIR catecholamine nanosensors were used to visualize micron-scale dopamine release hotspots in brain tissue [78]. Jeong et al. evolved serotonin-responsive DNA–SWCNT constructs from very large sequence libraries and demonstrated reversible responses in acute brain slices [79]. These examples show why dynamic sensing should be characterized by reversibility, response kinetics, localization, and interference testing rather than by acquisition rate alone. Recent single-molecule studies further resolve the kinetic behavior of neurotransmitter-responsive SWCNT sensors [80].

Intracellular analysis adds another layer of interpretation because probe motion and local optical conditions can change during acquisition. The nitric oxide study of Ulissi et al. illustrates the use of a model to relate time-dependent emission to analyte dynamics [55]. Such models can be valuable, but their assumptions and calibration domain should remain explicit. A stable long-term trace is most informative when controls demonstrate continued target responsiveness throughout the observation period, rather than only at its beginning.

### 6.5. Limitations

Optical sensing exchanges some electrical-interface constraints for requirements in excitation, collection, spectral discrimination, and calibration. NIR-compatible instrumentation must provide adequate sensitivity for the intended spatial and temporal resolution. Chirality mixtures can complicate spectral interpretation, while differences in dispersion or functionalization can change the contribution of individual emitting populations. Optical hardware and sample preparation are therefore part of the sensing system, not merely supporting accessories [9,62].

## 7. Molecular Recognition Engineering and Data-Driven Discovery

### 7.1. Conventional Recognition Elements

Comparisons of aptamer- and antibody-functionalized CNT-FETs illustrate how receptor dimensions can influence electrical coupling [10,11]. However, this should not be generalized into a universal ranking of aptamers over antibodies: affinity, conformational change, surface density, ionic conditions, and nonspecific adsorption all differ among target systems. The appropriate comparison is therefore transduction efficiency after immobilization under the intended assay conditions.

### 7.2. Corona Phase Molecular Recognition

Corona phase molecular recognition (CoPhMoRe) uses the structure adopted by an adsorbed polymer or related coating on a nanoparticle to recognize an analyte. On SWCNTs, this interface can couple recognition to NIR fluorescence. The assembled corona can create an arrangement of accessible regions and molecular interactions that is not represented by the coating molecule in free solution. This approach expands the recognition design space beyond pre-existing biological receptors, but it also makes the relationship between composition and function less obvious [6,22].

**Figure 6:**
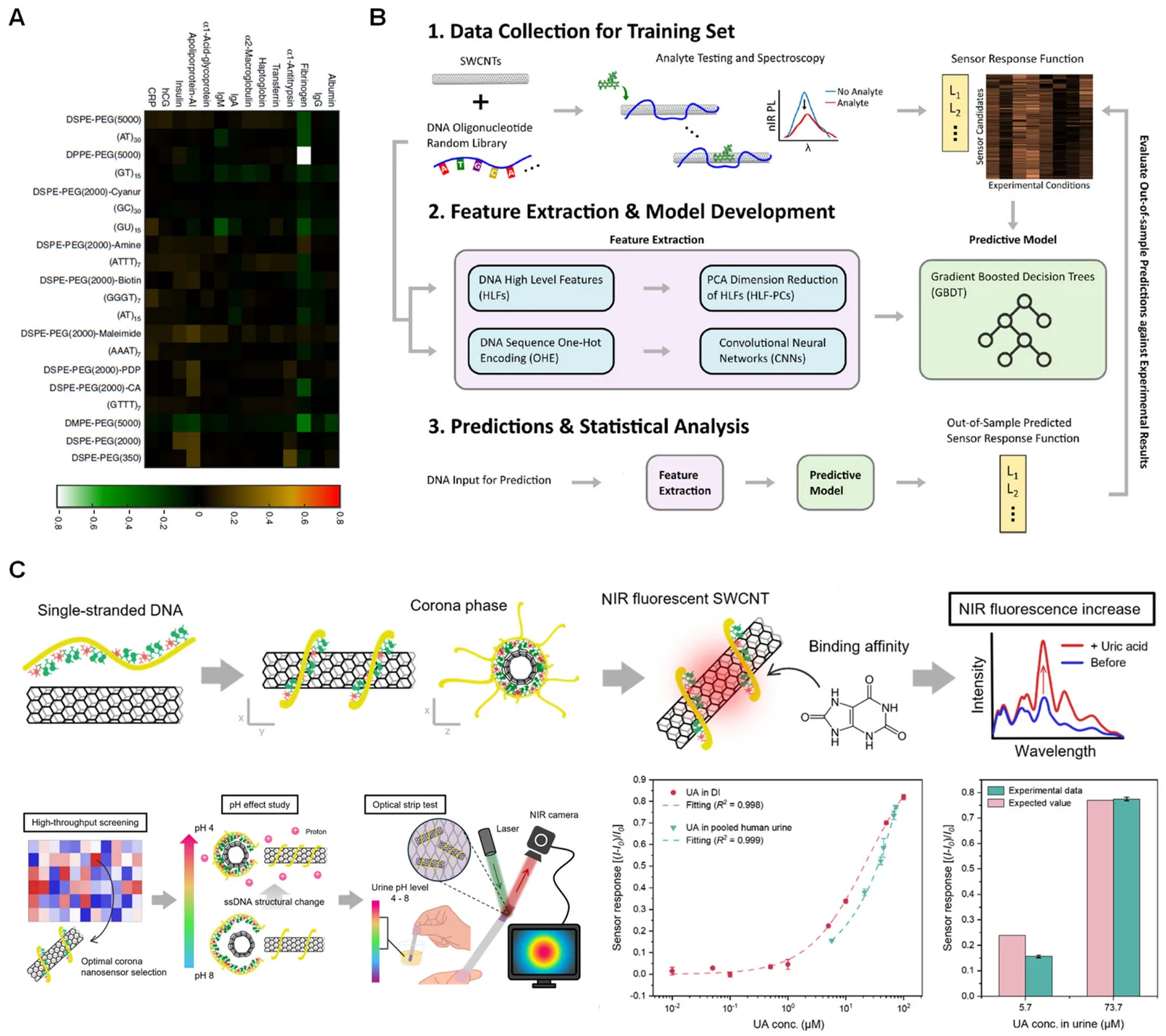
Experimental screening and data-driven discovery of SWCNT corona-phase recognition. (**A**) Protein-response heat map for a library of SWCNT corona phases. (**B**) Experimental and computational workflow linking DNA–SWCNT response measurements, sequence features, and predictive modeling. (**C**) Uric-acid recognition and assay development: corona assembly and recognition schematics (upper left and upper right), screening and pH optimization leading to an optical-strip assay (lower left), and solution-phase calibration in water and pooled human urine with a spiked-urine comparison (lower right). The calibration plots show solution-phase measurements rather than optical-strip performance. (**A**) Adapted from Bisker et al. [6] under CC BY 4.0; heat map rotated. (**B**) Reproduced from Gong et al. [22] under CC BY 4.0. (**C**) Adapted from Yoon et al. [7] under CC BY 3.0, published by the royal society of chemistry; selected components were cropped and rearranged.

Figure 6 brings together experimental corona screening (A), machine-learning-guided recognition discovery (B), and development of a urine-oriented assay (C). Bisker et al. screened 20 SWCNT corona phases against 14 proteins to identify a protein-responsive interface [6]. The study therefore provides more than a single-analyte demonstration: the candidate corona was selected from a response matrix and subsequently examined under competition and serum-containing conditions. The screening result and the subsequent selectivity tests should nevertheless be distinguished, because screening identifies a responsive interface whereas competition and matrix experiments test whether that response remains useful under more complex conditions. The response matrix in Figure 6A compares candidate coatings across the analyte panel, revealing a distinctive fibrinogen response for DPPE–PEG(5000)-wrapped SWCNTs. The authors then examined this candidate beyond the initial screen, including competition and serum-containing conditions. The study connects discovery to validation: the heat map identifies an interface worth pursuing, while the subsequent assays test the selectivity and context of that response. This is more informative than selecting a coating solely from its response to one isolated target.

Selectivity of a designed corona should be evaluated using a defined interference panel rather than by measuring the target alone. The panel should include structurally related molecules, likely coexisting biomolecules, and a matrix-only condition. Each interferent should be tested individually and, where relevant, in mixtures containing the target at a fixed concentration. Useful metrics include the target-to-interferent response ratio, the percentage of signal retained in the mixture relative to the target-only condition, the false-response level in interferent-only samples, and changes in the calibration curve. Competitive experiments should also examine whether increasing the concentration of a structurally related molecule suppresses, enhances, or does not affect the target response. A corona should be considered selective only when the target response remains distinguishable from the responses produced by related or competing species under the intended sample conditions. Bisker et al. followed this logic by screening multiple proteins and subsequently examining the selected corona under competition and serum-containing conditions [5]. Screening libraries can identify candidate interfaces, but selectivity must be confirmed using independent interference and mixed-sample experiments rather than inferred from a single-analyte response [21,22].

### 7.3. High-Throughput Screening

Large experimental libraries are useful because sequence, surface organization, and optical response are not linked by a simple universal rule. Changing a DNA sequence or polymer can alter its interaction with the nanotube and the accessibility of the target, while the response can also depend on chirality, analyte identity, pH, and preparation conditions. Screening should therefore explore a defined design space with controls that distinguish a selective response from a general change in fluorescence [6,22].

Figure 6C illustrates the transition from recognition screening to assay development [7]. A candidate selected for its response in buffer must still be evaluated for interference, matrix effects, and compatibility with the intended measurement format. These tests determine whether a screening result can support a practical assay.

### 7.4. Machine-Learning-Guided Discovery

Machine learning (ML) can extract relationships from response libraries and prioritize candidates for further testing. Its value depends on a clearly defined prediction task: predicting an optical response for a sequence under specified conditions is different from predicting selectivity in an unknown biofluid or classifying a patient sample. Training data, molecular descriptors, and the validation split must be chosen according to the task actually claimed [22].

Gong et al. assembled a library of 1408 DNA–SWCNT photoluminescence responses and used local sequence information together with higher-level descriptors to model recognition-dependent signals [22]. The workflow in Figure 6B separates sequence representation, model development, and out-of-sample evaluation. Predicted and measured responses were significantly correlated in six of eight experimental conditions and combining local and higher-level features improved performance over higher-level features alone. The study thus provides evidence that sequence information can guide candidate selection within a defined experimental domain. Variation between conditions also sets a limit on generalization: predictive recognition discovery is not equivalent to matrix-independent sensing or clinical classification.

The data-driven literature is now moving from response classification toward experimentally iterative optimization. Kelich et al. used machine-learning models trained on DNA-SWCNT response data to identify new serotonin-responsive sequences [81]. Subsequent work predicted serotonin responses across full fluorescence spectra rather than a single response value [82], while directed-evolution/ML screening improved both sensitivity and serotonin-over-dopamine selectivity across iterative rounds [83]. These studies support ML as a candidate-prioritization tool, but they also expose the dependence of prediction quality on the size, diversity, and independence of the experimental training set.

## 8. Manufacturability, Reproducibility, and Standardization

### 8.1. CNT Material Variability

Reproducible CNT biosensing requires traceability at three levels: the nanotube material, the fabricated electronic or optical element, and the biofunctionalized assay. Harmonized sample-preparation protocols and International Organization for Standardization (ISO) activities address material comparability and impurity characterization [84,85], whereas device studies add requirements for controlled network density, electrical stabilization, and analytical repeatability [86,87,88]. Reporting these levels separately helps locate whether variation originates in the CNT input, deposition process, recognition layer, packaging, or biological exposure.

The relative importance of these sources depends on the sensing modality, but they do not contribute equally across all devices. CNT purity and semiconducting fraction are particularly important for transistor and optical platforms because metallic CNTs, residual catalyst, and other impurities can increase leakage, background, or batch-to-batch variation [84,85]. For electrical devices, network density and morphology determine the number of conductive pathways and the relative contribution of individual CNT-FET junctions. Variations in network density can therefore change baseline conductance, sensitivity, and device-to-device dispersion [86,89]. Contact resistance and contact-related effects can also dominate the measured response, especially when the recognition layer modulates the metal–CNT junction rather than the nanotube channel itself [13]. Functionalization efficiency determines receptor coverage, surface accessibility, and the fraction of CNTs that remains exposed to nonspecific adsorption. Inconsistent functionalization can therefore produce variation even when the underlying CNT material and device geometry are nominally identical [31,56,57].

These parameters should be separated experimentally rather than treated as one combined fabrication variable. Material purity and chirality should be characterized before device fabrication, network density and morphology should be measured across the active area, and contact resistance should be evaluated independently of the biochemical response. Functionalization efficiency should be assessed using surface loading together with a functional accessibility or target-binding measurement. For analytical comparison, independently fabricated devices and independently prepared functionalization batches are more informative than repeated measurements on a single device [82,83,84,85,86].

The relevant material specification depends on the sensing modality. A semiconducting channel requires control of the electrically active CNT population, while an optical sensor depends on the emitting chiralities and their functionalized environment. Dispersion and processing can also influence bundling and the accessibility of the nanotube surface. Material characterization should therefore be linked to the response that the device is intended to produce, rather than assembled as a generic list of measurements detached from analytical performance [4,9].

Material identity should be traced through fabrication and functionalization to the final assay. Separately prepared dispersions and independently sourced material lots should be identified, and comparisons should determine whether calibration or background behavior changes with those inputs. This is particularly important when biological exposure creates an additional, variable coating [25]. Consistency at the raw-material stage does not guarantee consistency after processing, but it is necessary for locating the origin of downstream variation. Variation in analytical performance can originate from the CNT material, network morphology, contact geometry, or functionalization process. Material variability can affect background and electrical or optical activity; network density can alter baseline conductance and percolation; contact resistance can influence the measured current; and functionalization efficiency can change receptor loading and accessibility. These sources should therefore be evaluated separately using independent devices, fabrication batches, and functionalization preparations.

### 8.2. Network Morphology and Wafer-Scale Uniformity

Yahya et al. used airbrush deposition to prepare random SWCNT networks and examined their morphology and electrical behavior across device geometries [4]. Their fabrication sequence illustrates the deposition stage, while the resistivity comparison across channel lengths shows why layout belongs in process optimization. The study also incorporated antibody functionalization for human serum albumin detection in prepared analyte solutions. These results connect material deposition, device geometry, and biofunctionalization.

Manufacturing studies now provide quantitative benchmarks beyond selected laboratory devices. Liang et al. fabricated polymer-sorted semiconducting CNT biosensors across a 4-inch wafer and demonstrated uniform transistor performance for biomarker sensing [89]. Liu et al. subsequently reported an industrial-standard process for 4-inch CNT-FET wafers with performance variation below 6% within a wafer and below 9% between wafers, together with a plug-and-play portable readout [90]. These results shift the question from whether CNT biosensors can be fabricated to whether a process can repeatedly deliver devices within predefined electrical and analytical windows.

Baseline electrical characterization and analytical calibration address different forms of variability. Devices with similar resistance may still differ in recognition-layer coverage or target response. Conversely, a range of baseline values may be compatible with acceptable analytical behavior if the readout and calibration strategy handle it reproducibly. Distributions of both device properties and assay responses, together with predefined exclusion criteria, are therefore needed. Selecting only the most responsive devices obscures the manufacturing problem that the measurements are intended to resolve.

### 8.3. Scalable Fabrication and Assay Reproducibility

Scalable deposition and packaging must also survive wet operation and biofunctionalization. Ji et al. controlled random-network SWCNT density through chemical self-assembly, obtaining robust, uniform transistor films [86], while Molazemhosseini et al. demonstrated printed water-gated SWCNT transistors designed for rapid stabilization in biosensing conditions [87]. Li et al. further packaged uniform CNT-FET chips for aflatoxin B1 testing in real food samples and evaluated stability, repeatability, and blinded sample classification [88]. Together with wafer-scale fabrication [89,90], these studies connect film formation, liquid operation, packaging, and application-level reproducibility.

Zamzami et al. addressed a different dimension of reproducibility by testing 24 CNT-FET sensors from three fabrication batches, with groups exposed to saliva and S1-fortified saliva [26]. This comparison evaluates whether a target-associated response remains distinguishable across independently fabricated devices. It therefore complements the fabrication evidence discussed above: deposition and geometry define how devices are made, whereas batch testing examines the consistency of their analytical response. The sample size and grouping delimit the evidence; this comparison is not a measure of wafer-scale yield.

Li et al. examined CNT-FET commercialization as a problem spanning material control, device fabrication, functionalization, packaging, and validation [5]. Their industrialization framework connects fabrication and biointerface integration to reproducibility and application-relevant validation. The framework is conceptual, while the Yahya and Zamzami studies supply evidence for individual stages. At larger scale, separate yield measures for electrical function, recognition-layer preparation, and final assay performance are needed because success at one stage does not establish success at the next.

### 8.4. Packaging and Fluidic Interfacing

Wet and dry states require separate evaluation. A coating that performs immediately after preparation may respond differently after drying, storage, rehydration, or prolonged liquid exposure. Leakage, incomplete wetting, trapped air, and uncontrolled sample retention can also change the assay independently of the nanotube material. The assembled cartridge must be challenged through the sequence expected in use, including storage, sample loading, washing where applicable, and disposal or regeneration. The calibration must correspond to that sequence.

### 8.5. Metrology and Reporting Standards

Analytical reporting should include the blank definition, detection- and quantification-limit calculation where applicable, calibration range, uncertainty, response time, drift, and any regeneration procedure. The entire sample protocol should be specified, including dilution, pretreatment, capture, and washing. Recovery should be linked to the original sample concentration or amount, with enrichment factors and losses reported where relevant. These recommendations connect assay metrology to the sample-processing roles illustrated by the CNT plasma-extraction and capture platforms [27,38].

For data-driven readout, the calibration and validation datasets also belong in the metrology record. The sample or device unit used for splitting data should be clear, along with whether the algorithm was fixed before independent testing. An apparently reproducible model can otherwise reflect repeated measurements from the same preparation. The controlled response-prediction task in Gong et al. demonstrates why a model’s claimed domain must remain tied to its experimental dataset [22].

Environmental conditions should also be included in the measurement specification. Humidity can modify the adsorbed water layer, surface charge, analyte transport, and electrical response of CNT-based sensors. Shooshtari et al. demonstrated that humidity affects the gas-sensing performance of CNT sensors operated at room temperature, illustrating why environmental humidity should be controlled or reported when comparing CNT responses [91]. Although this study concerns gas sensing rather than biosensing, the result is relevant to the broader principle that CNT sensor performance depends on the measurement environment.

### 8.6. Analytical and Clinical Validation

Analytical validation should precede claims of clinical readiness. A sensor must first demonstrate reliable measurement in the intended matrix and workflow, including relevant interferences and variation among devices and samples. Clinical evaluation then asks a different question: whether the assay result supports the proposed decision in the intended population and setting. A favorable detection limit, an attractive device format, or a successful fortified-sample experiment cannot answer that question alone [5,26].

The studies considered here provide evidence for individual components of this progression: interface control, matrix response, device replication, imaging, or sample preparation. These contributions should be retained at their demonstrated level rather than combined rhetorically into proof of clinical deployment. Independent clinical or field validation consequently remains a required stage of development. The broader lesson is that translation depends on an evidence chain linking material consistency to a reliable result under the conditions in which that result will be used [5].

## 9. Outlook: Design Rules for Translation

### 9.1. Move from LOD-Centric to Robustness-Centric Benchmarking

Matrix tolerance, specificity, recovery, drift, reproducibility, and total time to result should therefore accompany sensitivity claims. Comparisons should distinguish buffer measurements, fortified matrices, pooled samples, individual-donor specimens, and prospective validation. These categories do not form interchangeable demonstrations of the same achievement. The donor-dependent coating profiles observed by García-Hevia et al. and the matrix-specific tests in optical and electrical sensors illustrate why biological variability belongs within performance assessment rather than outside it [7,25,26].

To facilitate comparison across CNT biosensor platforms, reported performance should be organized using a common set of fields: sensing modality and device architecture; target and recognition element; sample type and sample pretreatment; calibration range and matrix; limit of detection; response time; recovery or signal retention after matrix exposure; selectivity against relevant interferents; number of independently fabricated devices and biological samples; and storage or operational stability. A representative comparison of electrical, electrochemical, and optical CNT biosensor platforms using these fields is provided in Table 2. The limit of detection should be reported together with the calibration conditions and sample matrix because a value obtained in buffer, diluted sample, fortified sample, pooled specimen, or untreated individual specimen does not represent the same level of analytical validation.

For electrical CNT platforms, the comparison should additionally report gate or contact configuration, biasing conditions, baseline drift, hysteresis, and the number of independently prepared devices. For optical SWCNT platforms, the excitation and emission window, normalization method, fluorescence metric, photostability, optical background, and whether the experiment measures analyte recognition or probe localization should be specified. Response time should be defined consistently, and recovery should distinguish signal recovery after washing from analytical recovery of the target through a sample-preparation step. Reproducibility should be reported separately for repeated measurements, independently prepared sensors, different fabrication batches, and independent biological samples.

Because the reviewed studies use different targets, matrices, transduction mechanisms, and validation levels, we do not rank the platforms by a single numerical score. Instead, we recommend a standardized reporting framework that allows LOD, linear range, response time, recovery, reproducibility, and sample type to be interpreted together. This approach is more informative than comparing LOD values without considering matrix complexity, sample conditioning, device variability, or the level of validation [34,69,72,88].

Selectivity testing should also distinguish single-interferent experiments from multi-component interference testing. Testing one interferent at a time can identify specific vulnerabilities, but it may underestimate competitive adsorption and combined matrix effects. For sensors intended for biological samples, a more realistic evaluation should include the target in the presence of a defined mixture containing representative proteins, salts, metabolites, lipids, and other matrix components at physiologically relevant or sample-relevant concentrations. The response should be compared with target-only, interferent-mixture-only, and target-plus-interferent-mixture conditions. Reports should state the composition and concentration of the mixture, whether the components were tested simultaneously, the target recovery or signal retention in the mixture, and any changes in calibration slope, baseline, response time, or detection limit. A sensor that tolerates individual interferents may still show signal suppression, enhancement, or baseline drift when multiple components are present simultaneously.

### 9.2. Co-Design the Complete Stack

The studies reviewed here support development around the dominant limitation in the intended sample. Receptor presentation, biological adsorption, and sample conditioning affect different stages of the measurement chain and therefore require different interventions [24,25,27,38]. Reducing electronic noise cannot compensate for inaccessible binding sites, just as faster acquisition cannot compensate for poor target delivery. The complete system must be improved at the stage that limits the result.

### 9.3. Closed-Loop Sensor Development

The studies assembled in Figure 6 outline a progression from experimental recognition libraries through predictive candidate selection to evaluation in an application-relevant format [6,7,22]. Their combined significance is methodological: measured diversity supplies training evidence, models can prioritize candidates, and matrix-dependent experiments determine whether those candidates remain useful. These studies do not constitute a single experimentally completed closed loop, but they identify the components needed to build one.

### 9.4. Clinical and Real-World Validation

The validation question must match the intended claim. Fluorescence imaging establishes probe visibility and location, whereas analyte sensing requires a demonstrated relationship between the target and optical response. Likewise, recombinant-antigen detection in fortified saliva does not establish diagnostic accuracy in infected patients [8,9,26]. Computational predictions within a controlled response library also require independent validation before diagnostic generalization [22].

### 9.5. Final Perspective

CNT biosensors are most compelling when their nanoscale transduction is embedded within a reproducible sample-to-answer system. The constraints summarized in Figure 1 reappear throughout the experimental evidence: receptor geometry affects coupling, biological adsorption changes the interface, sample processing determines target access, and device preparation contributes to response variation. Together, these findings define a development pathway in which each stage requires its own evidence. Electrical and optical approaches offer complementary opportunities within this pathway, provided that their distinct mechanisms and validation requirements remain explicit [5,6,24,25,27,38].

Based on the evidence reviewed here, five priorities emerge for the next stage of CNT biosensor development. First, CNT materials and device structures should be made more reproducible by controlling CNT composition, network morphology, contact behavior, and fabrication variation. Second, recognition interfaces should be engineered for both molecular accessibility and resistance to nonspecific adsorption, with orientation, functionalization efficiency, and matrix exposure evaluated together. Third, sample handling should be treated as part of the sensor rather than as an external preprocessing step; separation, dilution, enrichment, delivery, and washing should be optimized according to their effects on recovery and matrix interference. Fourth, electrical and optical readouts should be evaluated using controls that distinguish target recognition from ionic, pH, adsorption, contact, optical-background, and other matrix-dependent effects. Fifth, analytical claims should be validated progressively from controlled buffer experiments to matrix-matched testing, independent devices and batches, individual biological specimens, and clinically relevant validation.

These priorities also define the boundary between different types of engineering problems. Improved CNT purity, network uniformity, contact design, surface chemistry, and receptor presentation can be addressed primarily through materials and device engineering. Transport limitations, target loss, and matrix interference require sample handling and system-level integration. In contrast, uncertainty about biological variability, clinical relevance, and diagnostic accuracy cannot be resolved by materials optimization alone and requires independent biological and clinical validation.

Across these studies, the central trend is a shift from isolated demonstrations of CNT sensitivity toward explicit control of the complete measurement chain. Electrical studies increasingly separate receptor geometry, ionic screening, passivation, contact effects, and device uniformity [11,12,31,44,45,57,89,90]. Optical studies have progressed from environment-sensitive NIR emission to synthetic recognition phases, intracellular and in vivo reporters, and data-driven sequence discovery [19,20,21,54,59,60,61,73,76,77,78,79,80,81,82,83,92]. The strongest translational studies are those that make these dependencies visible through independent devices and batches, matrix-matched controls, realistic sample processing, and validation that matches the intended claim [69,72,76,88]. Future CNT biosensors should therefore be compared by the completeness and reproducibility of their evidence chain, not by detection limit alone.

## Figures and Tables

**Table 1:** Matrix-dependent limitations, sensing consequences, and surface and sample-conditioning strategies for CNT biosensors.

Biological Matrix	Major Limitation	CNT Sensing Consequence	Surface/Sample-Conditioning Strategy	Representative Studies
Serum	High ionic strength; abundant albumin, immunoglobulins, lipids, and complement proteins; donor variability.	Charge screening; protein-corona formation; receptor masking; fouling-induced baseline drift and variable target access.	Compact or oriented receptors; hydrated antifouling layers; matrix-matched calibration; donor-to-donor testing.	[25,51,52]
Plasma	Abundant proteins, lipids, and complement components; composition affected by anticoagulant and separation protocol.	Charge screening; nonspecific adsorption; variable target recovery and matrix-dependent baseline changes.	Hydrated antifouling layers; standardized anticoagulant and separation protocols; recovery testing; matrix-matched calibration.	[25,27,30,53]
Whole blood	Blood cells, hemoglobin, platelets, clotting factors, high viscosity, and protein-rich background.	Obstructed sensor access; heterogeneous sample delivery; optical background; target loss during separation; surface fouling.	Plasma separation or filtration; controlled microfluidic delivery; cell-removal and recovery testing; antifouling interfaces; matrix-matched calibration.	[27,28,29,30]
Saliva	Variable mucins, proteins, and salts.	Nonspecific adsorption; variable background.	Compatible antifouling; matrix controls; defined sample conditioning.	[26,28,31]
Urine	Variable pH; interfering metabolites.	Altered fluorescence and selectivity.	Optimize DNA corona and pH; targeted pretreatment; matrix-matched calibration.	[7,32,33]
Intracellular/tissue	Probe localization; optical attenuation; local chemistry.	Location- and environment-dependent fluorescence.	Selective NIR probes; localization/background controls; compartment-specific calibration.	[19,20,21,54,55]

Note: Strategies represent a review synthesis; cited design studies do not establish validation in every listed matrix. Fortified-sample assays do not establish clinical diagnostic accuracy. Reference numbers follow the manuscript. Abbreviations: CNT, carbon nanotube; CNT-FET, carbon nanotube field-effect transistor; DNA, deoxyribonucleic acid; NIR, near-infrared; NO, nitric oxide.

**Table 2:** Standardized comparison of representative CNT biosensor platforms.

Ref.	Transduction	Target/Recognition Element	Sample Type	Pretreatment	LOD	Linear Range	Recovery/Signal Retention	Reproducibility	Validation Level
Yamada et al. [3]	SWCNT crossed-wire junction	*E. coli* K-12/antibody-mediated capture	Cultured bacterial sample	None reported	10^2^ CFU mL^−1^	NR	NR	NR	Cultured target
Zamzami et al. [26]	CNT-FET	SARS-CoV-2 S1/antibody	Fortified saliva	Saliva fortification	NR	NR	NR	24 devices from three batches	Fortified saliva; not clinical validation
Abdelhamied et al. [23]	Impedimetric AuNP/WO_3_/MWCNT electrode	Pathogenic bacteria/bacteriophage	Food sample	Sample-specific preparation	NR	NR	NR	NR	Food-matrix testing
Xu et al. [24]	Semiconducting CNT-FET	TEM-1/oriented BLIP2	Defined assay solution	Controlled electrolyte	NR	NR	NR	Comparison of two attachment positions	Mechanistic orientation study
Bisker et al. [21]	Optical SWCNT corona-phase sensor	Insulin/designed corona phase	Serum-containing buffer	Serum addition	NR	NR	Signal response reported; recovery NR	NR	Serum-containing analytical testing
Yoon et al. [7]	Optical SWCNT corona-phase sensor	Uric acid/designed corona phase	Water, pooled urine, spiked urine	Ascorbate-oxidase pretreatment	NR	NR	NR	NR	Matrix-relevant testing; not clinical validation
Chen et al. [65]	Semiconducting CNT-FET	Aβ40/Aβ42/aptamer	Human serum	None reported	Sub-femtomolar response reported	NR	NR	Device variation < 10%	Human-serum analytical testing
Albarghouthi et al. [45]	CNT transistor	Biomarker/antibody interface	1× PBS	Controlled electrolyte	NR	NR	NR	NR	Defined-electrolyte testing

Note: NR, not reported in the cited study. Values obtained in buffer, fortified matrices, pooled specimens, or processed samples should not be interpreted as equivalent to measurements in untreated individual clinical specimens. Recovery should be distinguished from signal recovery after washing, and reproducibility should specify whether it refers to repeated measurements, independently fabricated devices, different fabrication batches, or independent biological samples.

## Data Availability

No new data were created or analyzed in this study. Data sharing is not applicable to this article.
